# Sperm chemotaxis in marine species is optimal at physiological flow rates according theory of filament surfing

**DOI:** 10.1371/journal.pcbi.1008826

**Published:** 2021-04-12

**Authors:** Steffen Lange, Benjamin M. Friedrich

**Affiliations:** 1 HTW Dresden, Dresden, Germany; 2 Center for Advancing Electronics Dresden, TU Dresden, Germany; 3 Cluster of Excellence Physics of Life, TU Dresden, Germany; University of Illinois at Urbana-Champaign, UNITED STATES

## Abstract

Sperm of marine invertebrates have to find eggs cells in the ocean. Turbulent flows mix sperm and egg cells up to the millimeter scale; below this, active swimming and chemotaxis become important. Previous work addressed either turbulent mixing or chemotaxis in still water. Here, we present a general theory of sperm chemotaxis inside the smallest eddies of turbulent flow, where signaling molecules released by egg cells are spread into thin concentration filaments. Sperm cells ‘surf’ along these filaments towards the egg. External flows make filaments longer, but also thinner. These opposing effects set an optimal flow strength. The optimum predicted by our theory matches flow measurements in shallow coastal waters. Our theory quantitatively agrees with two previous fertilization experiments in Taylor-Couette chambers and provides a mechanistic understanding of these early experiments. ‘Surfing along concentration filaments’ could be a paradigm for navigation in complex environments in the presence of turbulent flow.

## Author summary

Many motile cells navigate in complex environments along concentration gradients of signaling molecules. This chemotaxis has been studied extensively both experimentally and theoretically, yet mostly for idealized conditions of perfect chemical gradients. But under physiological conditions, concentration fields are subject to distortions, e.g., by turbulent flows in the ocean. Pioneering experiments suggest that in species with external fertilization, chemotaxis of sperm cells towards the egg may even work better at an optimal flow strength compared to conditions of still water. Yet to date, the mechanistic cause for this optimum is not known. We present a general theory of chemotactic navigation in external flow. We characterize how external flow distorts concentration fields into long filaments, and show how chemotaxing cells can subsequently ‘surf’ along these filaments towards a chemoattractant source. Stronger flows make concentration filaments longer, but also thinner; together, these two counter-acting effects set an optimal flow strength. Beyond fertilization of marine invertebrates, we believe that ‘surfing along concentration filaments’ could be a more general paradigm, relevant also for the ecology of marine bacteria feeding on organic marine snow in the ocean, or chemotaxis inside multi-cellular organisms with internal flows.

## Introduction

Chemotaxis—the navigation of biological cells guided by chemical gradients—is crucial for bacterial foraging, neuronal development, immune responses, and sperm-egg encounter during fertilization [1–5]. Despite a century of research, most studies assumed perfect concentration gradients of signaling molecules. Yet, in natural environments, concentration fields of these chemoattractants are non-ideal, distorted e.g. by turbulent flows. An unusually accessible model system of such a cellular navigation is the chemotaxis of sperm cells in marine invertebrates with external fertilization. For fertilization, sperm cells of many species are known to employ chemotaxis to steer up concentration gradients of signaling molecules released by the egg. This sperm chemotaxis has been intensively studied for external fertilization of marine invertebrates, where sperm and egg cells are spawned directly into the sea [5–9]. In this case, sperm and egg cells become strongly diluted. Besides synchronized spawning [10, 11], sperm chemotaxis is important to enhance sperm-egg encounter rates [12]. The mechanism of sperm chemotaxis in marine invertebrates is well established theoretically [13, 14] and has been experimentally confirmed [15]: Sperm cells swim along helical paths **r**(*t*), while probing the surrounding concentration field *c*(**r**). A cellular signaling system rotates the helix axis **h** to align with the gradient **∇***c* at a rate proportional to a normalized gradient gradient strength |**∇***c*|/(*c* + *c*_*b*_) reflecting sensory adaption with sensitivity threshold *c*_*b*_ [16, 17].

**Fig 1:**
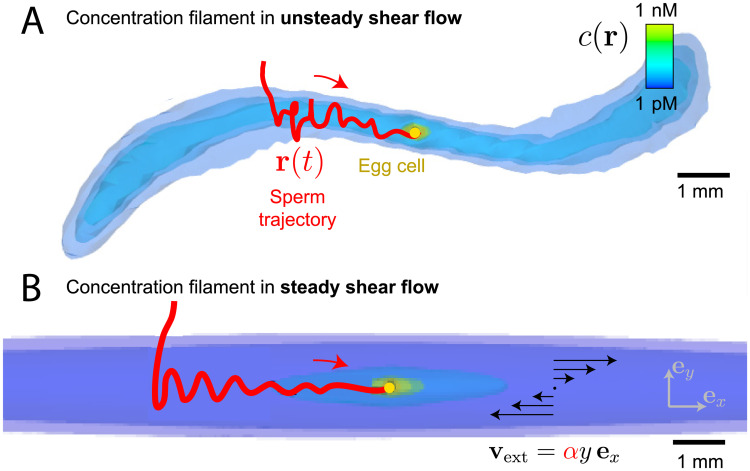
Sperm chemotaxis in external flow. (A) Simulated, three-dimensional concentration field *c*(**r**) of chemoattractant released from a freely-rotating, spherical egg (yellow sphere) suspended in unsteady shear flow as a model of small-scale turbulence. An exemplary simulated sperm cell (trajectory in red) finds the elongated concentration filament by chance and subsequently ‘surfs’ along the filament by chemotaxis. (B) Same as (A), but for the prototypical idealization of simple shear flow **v**_ext_(**r**) = *αy* **e**_*x*_ accounting for convection and co-rotation by the external flow. We obtain a generic form of the concentration filament, Eq (1), and characterize surfing along the filament analytically as a damped oscillation. Parameters correspond to sea urchin *A. punctuala*, assuming continuous release of chemoattractant at constant rate $\dot{Q}=0.46\;\text{fmol}\;{\text{min}}^{-1}$ for an exposure time *t*_max_ = 6 min. Constant shear rate *α* = 0.17 s^−1^ in (B), corresponding to root-mean-square shear rate of (A). Same color-code for concentration in (A) and (B), but different level sets. We use a generic theoretical description of helical sperm chemotaxis, see Methods and materials for details (helix radius *r*_0_ = 7 *μ*m not visible at length-scale of figure). Rotating views of the 3D images are provided as S1 and S2 Movies.

Previous work on sperm chemotaxis focused predominantly on idealized conditions of still water [9, 18]. However, natural habitats like the ocean are characterized by turbulent flow, which convects and co-rotates gametes and distorts concentration fields into filamentous plumes [7, 16, 19–23], see Fig 1A for illustration. Turbulence in typical spawning habitats of marine invertebrates has been characterized, e.g., in terms of local energy dissipation rates per mass *ϵ* = 10^−9^−10^−6^ m^2^s^−3^ [19, 22, 24–26] corresponding to typical shear rates *α* = 0.03−1 s^−1^, which are often similar to those in mammalian reproductive tracts [5]. Turbulent flow rapidly mixes sperm and egg cells, yet only down to the Kolmogorov length-scale *η*_Kol_ = (*ν*^3^/*ϵ*)^1/4^ = 1 − 10 mm (with kinematic viscosity *ν*). Previous predictions based on turbulent mixing [27] substantially underestimated fertilization probability *P*_fert_ [22, 28], since these early studies neglected active swimming and sperm chemotaxis inside the smallest eddies, whose size is comparable to the Kolmogorov length *η*_Kol_. At these small length-scales, the Reynolds number of the flow is below one, and gametes perceive turbulence as unsteady shear flow [24, 26] with a typical shear rate *α* set by the inverse of the Kolmogorov time $\tau_{\text{Kol}}=\sqrt{\nu /\epsilon }$. Intriguingly, fertilization experiments conducted at physiological shear rates hint at the existence of an optimal shear rate α* > 0, corresponding to an optimal turbulence strength ϵ* > 0, at which the fertilization probability *P*_fert_ was maximal [20, 25, 29]. Similar observations have been made in direct numerical simulations of bacterial chemotaxis [21]. Obvious biological effects can be ruled out as the origin of the optimum [19, 25], including flow damaging the gametes or sperm-egg bonds being broken by shear forces. Despite an early two-dimensional model [29], a physical explanation and quantitative understanding of the observed optimum is still missing [20, 22].

Here, we develop a theory of sperm chemotaxis in small-scale turbulence: As a prototypical model, we consider sperm chemotaxis in simple, three-dimensional shear flow, which convects and co-rotates sperm cells and distorts the chemoattractant field that surrounds the egg. We predict an optimal shear rate α* in simulations, as previously suggested by experiments [20, 25]. We provide a novel mechanistic explanation of this optimum from theory: We describe how external flow distorts concentration fields into slender filaments, and how sperm cells ‘surf’ along these filaments towards the concentration source, see Fig 1B. The optimum arises from the competition between accelerated spreading of the chemoattractant at increased flow, which enhances chemotaxis, and filaments becoming increasingly thinner, which impairs chemotaxis. We apply our theoretical description to two previous experiments on sperm chemotaxis, one with moderate flow, mimicking fertilization in shallow coastal waters [20], and one with strong turbulence, mimicking fertilization in the surf zone [25]. In both cases, simulation and theory match the experimental data, yet also prompt a partial re-interpretation of these early experiments: We infer a high background concentration of chemoattractant in these experiments, which actually masks the existence of an optimal flow strength for the experimental conditions used (in contrast to physiological spawning habitats where no relevant background concentration should be present). We propose that ‘surfing along concentration filaments’ could be a common navigation paradigm in natural habitats characterized by external flows, which is relevant for the last millimeters towards a source.

## Results

### Simulations: Optimal shear rate

**Fig 2:**
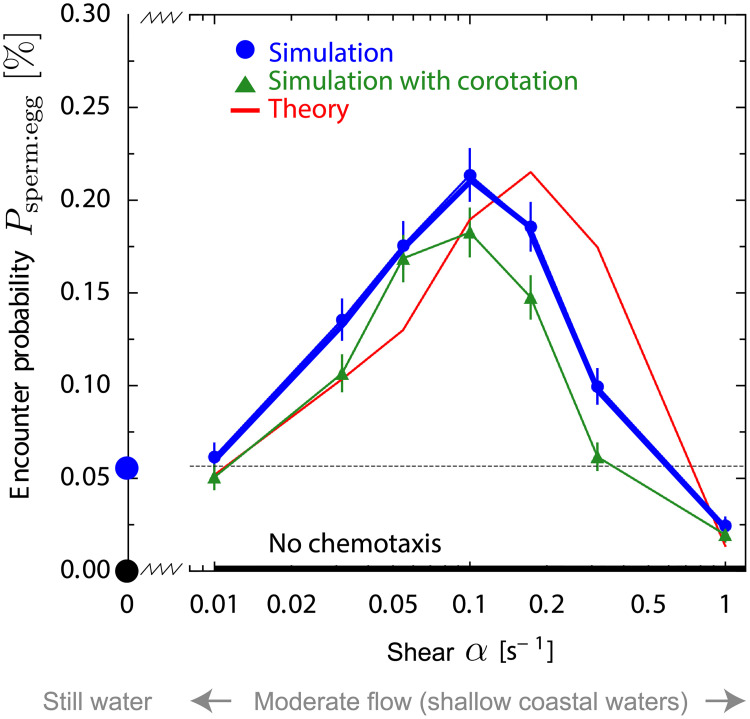
Sperm-egg-encounter probability displays maximum as function of shear rate in simulations for sea urchin sperm at physiological flow rates. Probability *P*_sperm:egg_(*α*) that a single sperm cell finds an egg as function of external shear rate *α*. Simulations account for flow-induced distortion of concentration fields into long filaments as well as convection and co-rotation of sperm cells by the flow (green triangles, mean ± SD). Without co-rotation results change only marginally (blue circles). Simulation results agree with predictions from our theory of *filament surfing* (red, presented below). Without sperm chemotaxis, the encounter probability is virtually zero (<10^−5^, black). Our theory has a single fit parameter, the flux of sperm cells arriving at the filament, *j*_out_ = 0.063 m^−2^s^−1^. This value matches in magnitude the limit *j*_out_ = *ρ*_egg_ *v*_*h*_/4 = 0.04 m^−2^s^−1^ for a ballistic swimmer with random initial conditions, see Sec E in S1 Text for details. Parameters as in Fig 1B.

We simulate sperm chemotaxis in a simple shear flow **v**_ext_(**r**) = *αy* **e**_*x*_, extending a generic theory of helical chemotaxis [14] by incorporating convection and co-rotation of sperm cells by the external fluid flow. In particular, for co-rotation by the flow the Jeffery equation [30, 31] is employed. We ask for encounters of sperm cells with a single egg that releases chemoattractant molecules, which establish a concentration field *c*(**x**, *t*) by convection and diffusion. By switching to a co-moving frame in which the egg is at rest. we may assume that the suspended egg is located at the origin **r** = **0** without loss of generality. We use a spherical periodic boundary at radius *r*_max_, which mimics an ensemble of eggs with density $\rho_{\text{egg}}={\left(4\pi r_{\text{max}}^{3}/3\right)}^{-1}$, and assume an exposure time *t*_max_. For turbulent flow, the exposure time would correspond to a typical time interval between subsequent intermittency events that re-mix sperm and egg cells and reset any concentration field of chemoattractant that might have been established in between. (Methods and materials provides details on simulation setup and extensive discussion of parameters). The resulting sperm-egg-encounter probability *P*_sperm:egg_ displays a maximum at an optimal shear rate α* ≈ 0.1 s^−1^, see Fig 2, which uses parameters for sea urchin *A. punctuala*. At the optimal shear rate α*, *P*_sperm:egg_ is 4-fold higher than without flow. Only for larger shear rates *α* > 0.3 s^−1^, chemotaxis becomes less effective than without flow and finally ineffective at very strong shear rate with *α* ≥ 1 s^−1^. Note that without chemotaxis, the encounter probability is 2-3 orders of magnitude smaller for the chosen parameters (not shown as not visible).

Surprisingly, the numerical results show that co-rotation of sperm cells is not necessary for the existence of an optimal shear rate as simulations without co-rotation yield very similar results, see Fig 2. Consequently, the existence of an optimal shear rate α* should be a consequence of the distortion of the concentration field by the flow. For simplicity, we thus focus on the case without co-rotation in the following (simulations with co-rotation are displayed in Figs A and C in S1 Text). Typically, shear flow generates long filaments, or plumes, of high concentration. Simulations show how sperm cells enter these filaments and ‘surf’ along them, see Fig 1B, with trajectories resembling a damped oscillation, see also Fig D in S1 Text. Damped oscillations occur when sperm cells move towards the egg, yet oscillations are amplified when sperm cells move away from the egg. The latter sometimes causes sperm cells swimming in the wrong direction to turn around, thus redirecting them towards the egg. In conclusion, sperm chemotaxis in external flows is a two-stage search problem [32] of first finding a concentration filament and subsequent chemotactic surfing along this filament towards the egg.

### Theory: Filament surfing

We develop a theory of sperm chemotaxis in filamentous concentration fields generated by simple shear flows. This theory describes surfing along filaments and allows to predict the sperm-egg-encounter probability, see Fig 2. We consider a simple shear flow **v**_ext_(**r**) = *αy* **e**_*x*_ and a spherical egg of radius *r*_egg_, without loss of generality located at **r** = **0**, releasing chemoattractant at a constant rate for a time *t*. The choice of the coordinate system corresponds to a co-moving frame in which the egg is at rest. Far from the source |**r**| ≫ *r*_egg_, the concentration field *c*(**r**, *t*) established by diffusion and convection takes a generic form, see Fig 1B for illustration,

$$\begin{array}{c} c\left(r,t\right)=c_{0}\exp(-k|x|)\exp(-\frac{{\left(y-y_{0}\right)}^{2}/a_{y}^{2}+z^{2}}{2\sigma^{2}})\;. \end{array}$$ (1)

Eq (1) describes a filament with exponential decay along its center line (*x*, *y*_0_(*x*, *t*), 0) and a Gaussian cross-sectional profile. We derived phenomenological power-laws for all parameters *c*_0_(*t*), *k*(*t*), *σ*(|*x*|, *t*), *a*_*y*_, and *y*_0_(*x*, *t*), see Sec C in S1 Text for details. Importantly, the effective decay length along the centerline of the concentration filaments increases with flow, 1/*k* ∼ *α*, while the effective diameter of the filaments decreases with flow, since the decay length *σ* away from the centerline of the filaments is independent of the flow while the base concentration *c*_0_ decreases with flow, *c*_0_ ∼ *α*^−1^. In this sense, the concentration filaments become longer and thinner with increasing shear rate *α*.

Sperm cells from marine invertebrates swim along helical paths, along which they measure the local concentration of chemoattractant [15]. This time-dependent concentration signal exhibits characteristic oscillations at the frequency of helical swimming, which encode direction and strength of a local concentration gradient. The concentration signal elicits a continuous steering response by which the helical swimming path aligns with the gradient. We generalize an effective equation for the alignment of the helix axis **h**(*t*) with the local gradient **∇***c*(**r**, (*t*)), previously derived for simple radial concentration fields [14],

$$\begin{array}{cc} \dot{\Psi } & =-v_{\varphi }\frac{\left|\nabla c\right|}{c+c_{b}}\text{sin}\Psi \;,\;\Psi =∢(\nabla c,h), \end{array}$$ (2)

with an effective response parameter *v*_*φ*_ of chemotactic signaling, to concentration filaments given by Eq (1), see Sec D in S1 Text for details. For a normalized distance *Y* of the sperm trajectory from the centerline of the concentration filament, we obtain a one-dimensional effective equation of motion which explains and quantifies filament surfing,

$$\begin{array}{c} \ddot{Y}=(\underset{∼\text{oscillator}}{\underset{︸}{-(1-{\dot{Y}}^{2})Y}}\pm \underset{∼\text{damping}}{\underset{︸}{\gamma \sqrt{\left(1-{\dot{Y}}^{2}\right)}\dot{Y}}})\underset{∼\text{dimmer}\;\text{switch}}{\underset{︸}{\frac{c}{c+c_{b}}}}\;. \end{array}$$ (3)

The single dimensionless parameter *γ* depends on the geometry of the concentration filament and chemotaxis parameters: *γ* decreases for longer and thinner filaments, while it increases with a rate of chemotactic re-orientation, see Sec D in S1 Text for details. To leading order, the effective equation of motion Eq (3) represents a damped harmonic oscillator. The corresponding frequency and damping ratio match the damped oscillation observed in simulations, see Fig 1 and Fig D in S1 Text. The strong gradient in the cross-section of the filament causes sperm cells to navigate towards the centerline of the filament. Yet, cells continuously pass through this centerline due to their finite chemotactic turning rate and consequently oscillate within the filament. The much weaker gradient along the concentration filament in Eq (1) damps this oscillation when sperm cells move towards the egg, and amplifies it when they move away.

The threshold *c*_*b*_ of sensory adaption limits chemotaxis to the part of the filament with concentration at least of the order of *c*_*b*_. This defines a cross-sectional area *A*(*x*), where *c*(**r**) ≥ *c_b_*, as well as circumference *S*(*x*), at each centerline position *x* of the filament. We decompose the search for the egg into an *outer search*, i.e., finding the concentration filament, and an *inner search*, i.e., surfing along the filament, see Sec E in S1 Text. For the outer search, we introduce the flux *j*_out_ of sperm cells arriving at the surface of the concentration filament and assume that *j*_out_ is approximately independent of the position *x* along the filament. Given that the egg has to be found within the exposure time *t*_max_, we also introduce the outer search time *t*_out_(*x*, *t*_max_)<*t*_max_ available to arrive at the filament at *x* as specified below. For the inner search, using the effective equation of motion, we compute the probability *p*_in_(*x*, *t*_max_) that a sperm cell entering the filament at position *x* reaches the egg within time *t*_max_. We also compute the conditional mean surfing time *t*_in_(*x*, *t*_max_), i.e., the average time successful sperm cells require to reach the egg after entering the filament at *x*. Correspondingly, we set the time for the outer search as *t*_out_(*x*, *t*_max_) = *t*_max_−*t*_in_(*x*, *t*_max_) for *p*_in_ > 0 (and *t*_out_ = 0 for *p*_in_ = 0). With these prerequisites, we can formulate a general formula for the sperm-egg encounter probability *P*_sperm:egg_ in the presence of shear flow (for a single sperm cell and egg cell density *ρ*_egg_)

$$\begin{array}{c} P_{\text{sperm:egg}}\approx \int_{-r_{\text{max}}}^{r_{\text{max}}}dx\;p_{\text{in}}\left(x,t_{\text{max}}\right)[A\left(x\right)\rho_{\text{egg}}+S\left(x\right)j_{\text{out}}t_{\text{out}}\left(x,t_{\text{max}}\right)]\;. \end{array}$$ (4)

The first term approximates the contribution from sperm cells that are initially within the filament. This contribution is negligible compared to the second term for low *ρ*_egg_ or large *t*_max_. The second term quantifies the contribution from sperm cells that successfully find the concentration filament and surf along it to the egg. The flux *j*_out_ can be determined either from a fit to full simulations or approximated as *j*_out_ = *ρ*_egg_ *v*_*h*_/4 by treating sperm cells outside the filament as ballistic swimmers with speed *v*_*h*_, see Sec E in S1 Text, both of which gives similar results. Moreover, the approximation of a ballistic swimming path outside of the filament is reasonable, as the persistence length of sperm swimming paths in the absence of chemoattractant cues was estimated as 3 − 25 mm [33], which is much greater than the diameter of concentration filaments.

Note that for the chosen parameters, the volume $V_{\text{tot}}=\int_{-\infty }^{\infty }dx\;A\left(x\right)$ of the filament (and its surface area $\int_{-\infty }^{\infty }dx\;S\left(x\right)$) increases monotonically with shear rate *α*. Hence, the optimal α* is not explained by a flow-dependent ‘chemotactic volume’ *V*_tot_. Instead, the optimum emerges from two effects related to filament surfing, which reduce *p*_in_ and *t*_out_ in Eq (4) at high *α*: First, when the filament is too thin at the entry point *x* to enable the first oscillation, the sperm cells simply pass through the filament, which corresponds to low or vanishing probability *p*_in_. Second, if the time required to surf from the entry point *x* to the egg is too long, which corresponds to low or vanishing *t*_out_, the sperm cells will not reach the egg during the exposure time *t*_max_. Higher shear rates generate longer and thinner filaments, which aggravates both effects.

Comparison of full simulations and the theoretical prediction Eq (4) shows good agreement, see Fig 2. This agreement strongly suggests that the optimal shear rate α* originates from two competing effects: Higher shear flow spreads the chemoattractant faster, which facilitates sperm navigation to the egg, but results in longer and thinner filaments, which impairs chemotactic filament surfing. The value of the optimal shear rate α* could be adjusted to a different value by re-scaling the biological parameters that involve a time-scale such as the diffusion coefficient and release rate of chemoattractant or the swimming and chemotactic re-orientation speed of sperm cells, see Sec D in S1 Text.

According to our theory, the presence of an optimal flow strength is a generic feature at low egg densities and relatively long exposure times. Amplitude and position of the peak of the sperm-egg-encounter probability *P*_sperm:egg_(α*) depend on chosen parameters. Our theory allows to compute *P*_sperm:egg_(*α*) for any given set of parameters and thus the parameter-dependency of the optimal shear rate α* can be explored. A numerical parameter study is presented in Sec I in S1 Text, which demonstrates the robustness of the existence of an optimal shear rate under parameter variation. In short, a higher egg density *ρ*_egg_ and longer exposure time *t*_max_ increase the absolute amplitude *P*_fert_(α*) of this peak, while α* stays almost constant. A high sensitivity threshold *c*_*b*_ of chemotactic signaling, which is formally analogous to a high background concentration of chemoattractant, reduces the relative amplitude *P*_sperm:egg_(α*)/*P*_sperm:egg_(*α* = 0) of the peak. Significantly shorter exposure time *t*_max_ or higher egg density *ρ*_egg_ reduce *p*_in_ by effectively cutting off the outer parts of the filament. Note that the optimal shear rate α* is slightly smaller in simulations, compared to the theory. Inspection of simulated trajectories suggest that this is due to sperm cells, which miss the egg at least once while surfing along the filament, which increases the mean surfing time *t*_in_.

### Comparison with experiments

Previous experiments measured the fraction of fertilized eggs *P*_fert_ for an exposure time *t*_max_ of mixed sperm and egg cells. This fraction directly relates to the encounter probability *P*_sperm:egg_ by fertilization kinetics [34, 35] when the respective densities of sperm and egg cells, *ρ*_sperm_ and *ρ*_egg_, are known

$$\begin{array}{c} P_{\text{fert}}\left(t_{\text{max}}\right)=1-\exp(-p_{\text{f}}P_{\text{sperm:egg}}\left(t_{\text{max}}\right)\frac{\rho_{\text{sperm}}}{\rho_{\text{egg}}})\;. \end{array}$$ (5)

The fertilizability *p*_f_ is the probability that a sperm-egg-encounter results in successful fertilization. Note that a local maximum of the encounter probability *P*_sperm:egg_ at some optimal shear rate α* automatically gives a local maximum of the fertilization probability *P*_fert_. In particular, the density of sperm only alters the absolute value of *P*_fert_ across all shear rates but not the existence and value of an optimal shear rate α*.

#### Moderate shear

**Fig 3:**
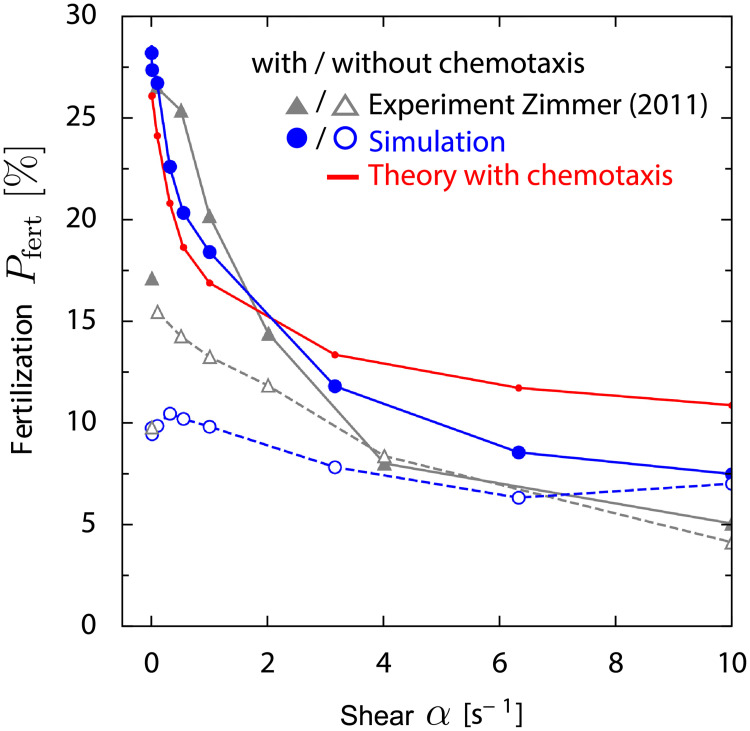
Comparison to experiment at moderate shear rates and short exposure time. Fertilization probability *P*_fert_(*α*) that an egg becomes fertilized as function of external shear rate *α* from previous experiments with red abalone *H. rufescens* gametes in a Taylor-Couette chamber (filled gray triangles: with chemotaxis, open gray triangles: inhibited chemotaxis; for *α* = 0 s^−1^ a different experimental protocol was used) [20] and our corresponding simulations (filled blue circles: with chemotaxis, open blue circles: without chemotaxis, mean ± SD). We find reasonable agreement using a single fit parameter, fertilizability *p*_f_ ≈ 60%, which characterizes the fraction of sperm-egg encounters that result in successful fertilization, see Eq (5). From the experimental protocol, we estimate a background concentration *c*_bg_ ∼ 4 nM of chemoattractant. While our theory of filament surfing does not directly apply due to this high background concentration, a near-field estimate (red line) yields a similar decay of fertilization probability as function of shear rate *α*. The single fit parameter of the theory, *j*_out_ = 4.8 ⋅ 10^3^ m^−2^s^−1^, is again consistent with the limit *j*_out_ = *ρ*_egg_ *v*_*h*_/4 = 7.5 ⋅ 10^3^ m^−2^s^−1^ of a ballistic swimmer with random initial conditions (note the the higher value of *j*_out_ compared to Fig 2 due to higher egg density). For simplicity, simulations do not account for co-rotation of sperm cells, see Fig A in S1 Text for results with co-rotation.

In a previous experiment by Zimmer and Riffell, fertilization was studied for red abalone *H. rufescens* in a Taylor-Couette chamber for moderate shear rate *α*, mimicking flow conditions in their natural spawning habitat [19, 20]. The measured fertilization probability decreased with increasing *α* > 0, both for normal chemotaxis and a case of chemically inhibited chemotaxis, see Fig 3 for a reproduction of the original data ([20], Fig. 5c). At low shear rate, the measured fertilization probability is twice as high with chemotaxis than without, while there was little difference at high shear rates. This suggests that the performance of sperm chemotaxis is reduced at high shear rates. We performed simulations of sperm chemotaxis in external flow, using parameters that match the specific experimental setup of [19, 20], see Sec G in S1 Text. Specifically, the time span between preparation of the egg suspension and the actual fertilization experiment results in a background concentration of chemotattractant, which we estimate as *c*_bg_ ∼ 4 nM, i.e., several orders of magnitude larger than the threshold of sensory adaption *c*_*b*_, and account for in the simulations. We compare results of these simulations and the experiments, using fertilizability *p*_f_ as single fit parameter, see Fig 3. We find good agreement for the case with normal chemotaxis, and reasonable agreement for the case of inhibited chemotaxis (potentially due to residual chemotaxis in the latter case). An exception is the data point at *α* = 0 s^−1^. In fact, a different experimental protocol was used for this data point, corresponding to different initial mixing of sperm and egg cells, which is not modeled in the simulations. In Fig 3, we neglected co-rotation of sperm cells for simplicity. We find similar results if we account for co-rotation, except for the highest shear rates, where fertilization probability is reduced, see Fig A in S1 Text. For simplicity, a shear rate dependent chemokinesis as suggested by [19, 20], i.e., regulation of sperm swimming speed, is not included in the model, as preliminary simulations suggest that this changes results only slightly. In our comparison, we focused on the case of low sperm density considered in [19, 20], thereby avoiding confounding effects of sperm-sperm interactions and reduced fertilization rates due to polyspermy at high sperm densities [36, 37].

The absence of an optimal shear rate α* is caused by the high background concentration *c*_bg_ in the experiment: Due to *c*_bg_, the part of the filament with sufficiently high concentration *c*(**r**) ≳ *c_b_* + *c_bg_* is situated only in the vicinity of the egg and has an approximately spherical shape. While our far-field theory of filament surfing does not directly apply to this special near-field case, a simple estimate for *p*_in_ and *t*_out_ assuming straight sperm trajectories aligned with the local concentration gradient inside the plume, see Sec E in S1 Text, yields a similar decay of fertilization probability, see Fig 3. The fitted flux of sperm cells into the concentration plume *j*_out_ = 4.8 ⋅ 10^3^ m^−2^s^−1^ is consistent with the limit *j*_out_ = *ρ*_egg_ *v*_*h*_/4 = 7.5 ⋅ 10^3^ m^−2^s^−1^ for a ballistic swimmer. This validates our interpretation of chemotaxis in external shear as a two-stage search, consisting of blind random search for a chemotactic volume and subsequent navigation inside this volume. We emphasize that the high background concentration of chemoattractant, which we reconstruct for these experiments, has a strong effect on the fertilization dynamics. Such high background concentrations are unlikely to be encountered in natural habitats, where eggs are spawned and consequently diluted in the open water.

#### Strong flows

**Fig 4:**
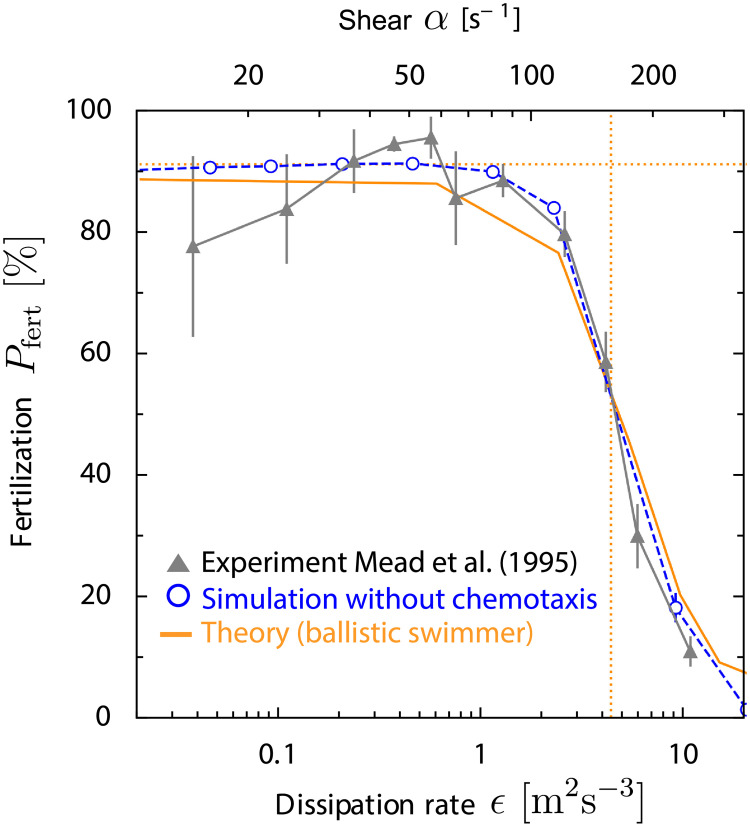
Fertilization in strong flows and high egg density. Previous measurements of fertilization probability *P*_fert_(*ϵ*) for sea urchin *S. purpuratus* at strong turbulence, characterized by density-normalized dissipation rate *ϵ* (filled gray triangles) [25, 38] and our corresponding simulations *P*_fert_(*α*) as function of shear rate *α* (open blue circles, mean ± SD) match well, using a single fit parameter *a* = 0.075 that relates dissipation rate *ϵ* and typical shear rate *α* (using the known relationship $\alpha \left(\epsilon \right)=a\sqrt{\epsilon /\nu }$ [24, 26]). Both simulation and experiment are well captured by a minimal theory of a ballistic swimmer in simple shear flow (red), see Sec A in S1 Text. Fertilization probability *P*_fert_ rapidly drops above a characteristic flow strength *α* > 100 s^−1^, which is consistent with a scale estimate *α* = 2*πv*_*h*_/(0.1*r*_egg_) (vertical dotted line). At these high shear rates, active swimming becomes negligible compared to convection. The case of low shear rates is well described by the limit case of a ballistic swimmer in the absence of flow *α* = 0 s^−1^ (dotted horizontal line, Eq (5) with *P*_sperm:egg_(*t*) = 1 − exp(−*qt*) and rate $q=\pi r_{\text{egg}}^{2}v_{h}\rho_{\text{egg}}$). The fertilizability *p*_f_ = 10% is obtained from an independent experiment [25], see Fig B in S1 Text. From the experimental protocol, we estimate a high background concentration *c*_bg_ = 500 − 4000 nM of chemoattractant, which renders sperm chemotaxis ineffective. Corresponding results for simulations with co-rotation are shown in Fig C in S1 Text.

Mead and Denny studied fertilization in the sea urchin *S. purpuratus* in turbulent flow, mimicking physiological conditions in the oceanic surf zone [25, 38, 39]. The measured fertilization probability slightly increased as function of turbulence strength, quantified in terms of local dissipation rate *ϵ*, and decreased rapidly at larger dissipation rate *ϵ* > 1 m^2^s^−3^, see Fig 4 for a reproduction of the original data (taken from Fig. 3 of [38], representing a re-calibration of data from Fig. 5 of [25]). We determined fertilization probability *P*_fert_ in simple shear flow from simulations, using parameters that match the specific experimental setup, see Sec G in S1 Text. For the experiments by Mead and Denny, we estimate a high background concentration of chemoattractant *c*_bg_ = 500 − 4000 nM, which renders sperm chemotaxis ineffective, which is thus neglected in the simulations. Fully developed turbulence is characterized by a spectrum of local shear rates, with a characteristic shear rate *α* related to the dissipation rate by $\alpha \left(∊\right)=a\sqrt{∊/\nu }$ with proportionality factor *a* [24, 26]. In the simulations, we assume a simple shear flow **v**_ext_(**r**) = *αy* **e**_*x*_, and determine *a* = 0.075 by a single-parameter fit, see Fig 4. For sake of simplicity, co-rotation of sperm cells is neglected. Results with co-rotation are qualitatively very similar, yet the fertilization probability *P*_fert_ drops at a smaller shear rate *α* and thus yields a smaller fit parameter *a* = 0.023, see Fig C in S1 Text. Note that these fits for *a* are smaller than values commonly used in the literature *a* ∼ 0.15 − 1.8 [24, 40, 41]. Nevertheless, our minimal model already reproduces the experimentally observed characteristic drop in fertilization probability *P*_fert_(*ϵ*) at high flow rates, implying that this is a robust, general feature.

We can capture the functional dependence of the fertilization probability *P*_fert_ observed in both experiment and simulations by a minimal theory of a ballistic swimmer in simple shear flow, see Fig 4 and Sec A in S1 Text. In particular, for small shear rate *α*, *P*_fert_ is close to the asymptotic limit *P*_fert_(*α* = 0) of a ballistic swimmer without flow. The drop of *P*_fert_ at strong flow can be estimated from a simple scaling argument: At high shear rate *α* ≥ *v*_*h*_/*r*_egg_, the active swimming of sperm cells is negligible compared to the external flow, except in the direct vicinity of the egg. This vicinity is set by a characteristic distance *δ* ∼ 0.1*r*_egg_ from the egg, up to which the flux of sperm cells is elevated (due to the geometry of the streamlines around the egg). To reach the egg, these sperm cells have to traverse a distance ∼*δ* within the typical time *t*_*δ*_ ∼ 2*π*/*α* that corresponding streamlines spend in the vicinity of the egg (time for half rotation of the egg). Thus, the characteristic flow strength at which *P*_fert_ drops can be estimated as *α* ∼ 2*πv*_*h*_/*δ*, see Fig 4.

For Fig 4, we obtain the fertilizability *p*_f_ ≈ 10% from an independent experiment in the absence of flow [25], which is well described by the fertilization kinetics, Eq (5), see Fig B in S1 Text. This *p*_f_ is larger than a value *p*_f_ = 3.4% previously reported for sea urchin *S. franciscanus* [34, 35]. However, these previous experiments were conducted at much higher sperm densities, where sperm-sperm interactions and polyspermy [36, 37] may reduce the fertilization probability. The estimated fertilizability for sea urchin is smaller than our estimate for red abalone *p*_f_ = 60%, which is expected due to the jelly coat of sea urchin eggs: For red abalone, sperm cells are considered to arrive directly on the egg surface, whereas for sea urchin, sperm cells are considered to arrive at a jelly coat surrounding the egg, which sperm cells have to penetrate before fertilization.

## Discussion

**Fig 5:**
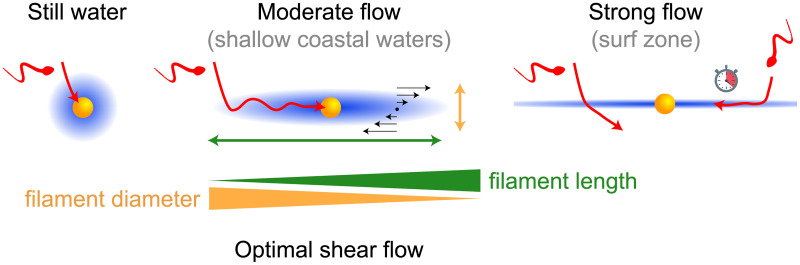
Proposed mechanism explaining optimal flow strength for sperm chemotaxis. Egg cells (yellow) release signaling molecules (blue) that guide sperm cells of marine species with external fertilization (red). External flows (black arrows) stretch concentration gradients into millimeter-long filaments. If sperm cells encounter such filaments, they can “surf” by chemotaxis towards the egg. In strong flows, however, sperm cells may fail to follow the filament after encounter, because the effective diameter of filaments is too small. Additionally, in very long filaments, sperm cell may not reach the egg within the sperm-egg exposure time (which is set by the lifetime of the smallest eddies for turbulent flow). Thus, it is not the total volume of the chemoattractant plume that determines fertilization success, but the geometric shape of filaments. The competition between increasing filament length, which favors sperm-egg encounters, and decreasing filament diameter, which jeopardizes filament surfing, sets an optimal flow strength that maximizes sperm-egg encounters. The optimal flow strength predicted by our theory matches physiological flow strengths in typical habitats.

We presented a general theory of sperm chemotaxis at small-scale turbulence, using marine sperm chemotaxis in physiological shear flow as prototypical example. We predict that sperm chemotaxis performs better in physiological flows as compared to conditions of still water. Our theory provides a novel phenomenological description of concentration filaments shaped by external flow, and describes how sperm cells surf along these filaments in terms of damped oscillations. Extensive simulations show that fertilization success becomes maximal at an optimal flow strength. We explain the existence of this optimal flow strength as the result of a competition between a faster built-up of concentration gradients in the presence of flow, and the disadvantageous distortion of concentration fields into increasingly thinner concentration filaments at increased flow rates, see also Fig 5. The optimal flow rate predicted by our theory matches typical flow strengths in typical spawning habitats in shallow coastal waters [19, 22, 24–26]. The maximal sperm-egg encounter probability at the optimal flow rate depends strongly on egg density and sperm-egg exposure time, see parameter study in Sec I in S1 Text. In contrast, the optimal flow rate α* as predicted by our theory is largely independent of egg density, sperm-egg exposure time, and other parameters.

In our simulations, we considered a constant sperm-egg exposure time, independent of flow strength, following the experimental protocol from [19, 20, 25, 38]. For fully developed turbulence in natural habitats, the life time of the smallest eddies sets an effective sperm-egg exposure time, as the turn-over of small-scale eddies resets local concentration fields surrounding the egg. As a consequence, the sperm-egg exposure time decreases with increasing flow strength (approximately, *t*_max_ ≈ 25/*α* ∼ *ϵ*^−1/2^, using the decay time scale of corresponding Burger vortices, see Sec H in S1 Text). This provides yet a third effect that reduces the success of sperm chemotaxis in strong turbulence. While the steady shear flow is a minimal model, we expect more realistic turbulence simulations to yield similar results at the relevant scales based on simulations with an unsteady shear flow, as shown in Fig 1A. Although, the shear rate is rather large *α* = 0.17 s^−1^, i.e., almost twice as large as the shear rate at the optimum, concentration filaments are only slightly bent by the rotational diffusion of the shear axis in unsteady shear flow, and we consistently find that sperm cells surf along slender concentration filaments, as exemplified in Fig 1A.

Our numerical simulations quantitatively account for previous fertilization experiments in Taylor-Couette chambers. These experiments impressively demonstrated the reduction of fertilization success at high flow rates and hinted at the existence of an optimal flow rate, which motivated our theoretical study. Our theoretical analysis highlights two subtleties in the interpretation of these early experiments, i.e., a high background concentration of chemoattractant and possibly insufficient mixing of sperm and egg cells in the absence of flow, both of which can confound an existing optimum. Nonetheless, we are indebted to this pioneering work and can now predict conditions, under which an optimal flow strength is expected. This can aid the rational design of future experiments. While a direct experimental observation of filament surfing is pending, recent 3D tracking experiments of sea urchin sperm cells navigating in axially symmetric chemoattractant landscapes gave intriguing anecdotal evidence how these cells first found the centerline of these concentration filaments and subsequently moved parallel to this centerline [15]. While our numerical simulations consider a specific mechanism of sperm chemotaxis along helical paths, our analytical theory is more general and applies in particular to any chemotaxis strategy for which the swimming direction gradually aligns to the local gradient direction. This suggests that the presence of an optimal flow strength could be a general phenomenon.

We expect that our findings of two-stage chemotactic search, comprising finding a filament and subsequent surfing along this filament, could be also relevant for foraging of bacteria and plankton: Nutrient patches are stirred by turbulent flow into networks of thin filaments, in which these organisms have to navigate for optimal uptake. Finding sinking marine snow from whose surface nutrients dissolve bears resemblance to finding egg cells which release chemoattractant. These organisms play an important role for oceanic ecosystems [21, 26, 42–48]. While our theory addresses the experimentally more accessible model system of external fertilization as employed by marine invertebrates [6], chemotaxis in external flows is relevant also for internal fertilization, where sperm cells navigate complex environments [49, 50], likely guided by both chemotaxis [5] and rheotaxis [51–53]. We emphasize that rheotaxis and chemotaxis in the presence of external flow as considered here rely on different physical mechanisms, despite formal similarities, such as active swimming upstream an external flow. While rheotaxis relies on the co-rotation of active swimmers, we found co-rotation to be dispensable for successful chemotactic navigation, with upstream swimming arising solely from chemotactic alignment to concentration filaments shaped by flow.

More generally, we characterized sperm chemotaxis in external flow as a combination of random exploration, followed by local gradient ascent, which corroborates a general paradigm for cellular and animal search behavior [54]. The minimalistic information processing capabilities of sperm cells (comparable to that of a single neuron [9]) can inspire biomimetic navigation strategies for artificial microswimmers with limited information processing capabilities intended for navigation in dynamic and disordered environments [55, 56].

## Methods and materials

The encounter probability *P*_sperm:egg_ is computed numerically by simulating sperm trajectories **r**(*t*) in the presence of both a concentration field *c*(**r**) of chemoattractant and an external fluid flow field **v**_ext_(**r**) according to equations of motion for **r**(*t*), see Sec B in S1 Text. These equations extend a previous, experimentally confirmed theory of sperm chemotaxis along helical paths [14, 15] by incorporating convection and co-rotation of cells by the external flow. For co-rotation, we employ Jeffery equation for prolate spheroids [30, 31] by assigning sperm cells an effective aspect ratio *g* = 5. For the shear rates considered here, the effect of external flow on sperm flagellar beat patterns is negligible [57]. Each sperm cell is simulated for an exposure time *t*_max_, which is set by the protocol of the corresponding experiment, or until it hits the surface of the egg.

As external flow, we assume a simple shear flow around a freely-rotating spherical egg, see Sec A in S1 Text. Throughout, we consider the co-moving frame of the egg allowing us to assume that the egg is at the origin **r** = 0. The concentration field is established by diffusion and convection from the egg releasing chemoattractant at a constant rate. We consider the reference case of a static concentration field corresponding to a chemoattractant release time equal to exposure time *t*_max_. Note that the exposure time *t*_max_ may also be estimated by the decay time scale of a Burger vortex *t*_max_ ∼ 25/*α*, see Sec H in S1 Text. To account for an ensemble of eggs at density *ρ*_egg_, we consider a single egg with radius *r*_egg_ at the origin **r** = **0** and a spherical domain with radius *r*_max_ = (4*πρ*_egg_/3)^−1/3^ and appropriate periodic boundary conditions: Initially, sperm cell positions **r**(*r*_egg_ ≤ |**r**| ≤ *r*_max_) and directions of the helix axis **h** are uniformly distributed, representing the distribution after initial turbulent mixing of egg and sperm cells. If sperm cells leave the simulation domain, they re-enter with random new initial conditions **r** and **h** with |**r**| = *r*_max_, whose distribution *P_b_*(**r**, **h**) is defined by the theoretical in-flux of cells due to active swimming and convection

$$\begin{array}{c} P_{b}(r,h)∼-p_{\text{sperm}}(r,h)[(v_{\text{ext}}(r)+v_{h}h)\cdot e_{r}(r)] \end{array}$$ (6)

with uniform and isotropic distribution of sperm cells *p*_sperm_ (**r**, **h**). In principle, co-rotation of non-spherical particles by shear flow leads to a non-uniform distribution of directions **h**, see analytic solutions in Sec F in S1 Text, but the effect on simulation results is negligible.

Parameters for Fig 2 were chosen to closely match conditions of *A. punctuala* sea urchin in their natural spawning habitat at low egg density *ρ*_egg_ and relatively long exposure times *t*_max_. Parameters for Figs 3 and 4 are chosen to match the experiments by Zimmer and Riffell [19, 20] and Mead and Denny [25], respectively. For further details on simulations and extensive discussion of parameters used for each scenario, see Sec G and H in S1 Text. Finally, error bars for simulation results represent simple standard deviation (SD) of the corresponding binomial distribution. Error bars are smaller than symbol sizes in some cases.

## Supporting information

S1 Text Supporting figures and tables. The Supporting Information text provides details on numerical methods, a derivation of our analytical theory, a discussion of parameter values, and a systematic study on the effect of model parameters (including egg density and sperm-egg exposure time).
(PDF)

S1 Movie Supporting information movie S1 provides rotating view of Fig 1A. (MP4)

S2 Movie Supporting information movie S2 provides rotating view of Fig 1B. (MP4)
