# Supplementary material for: Sperm chemotaxis in marine species is optimal at physiological flow rates according theory of filament surfing
Source: PLoS Comput Biol. 2021 Apr 12;17(4):e1008826. doi: 10.1371/journal.pcbi.1008826 (PMC8041200; doi:10.1371/journal.pcbi.1008826)
Supplement: S1 Text — The Supporting Information text provides details on numerical methods, a derivation of our analytical theory, a discussion of parameter values, and a systematic study on the effect of model parameters (including egg density and sperm-egg exposure time). (PDF) [file pcbi.1008826.s001.pdf]

Supporting Information: Sperm chemotaxis in marine species is optimal at physiological flow rates

Steffen Lange[1,2], Benjamin M. Friedrich[2,3]

1 HTW Dresden, Dresden, Germany
2 Center for Advancing Electronics Dresden, TU Dresden, Germany
3 Cluster of Excellence Physics of Life, TU Dresden, Germany

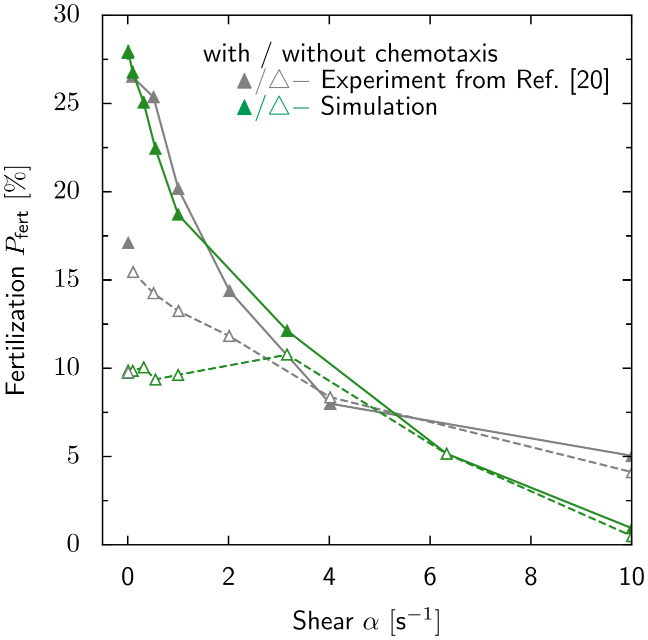

Fig A. Fertilization probability with co-rotation. Fertilization probability $P_{\mathrm{fert}}(\alpha)$ as in Fig 3 but with simulations taking into account co-rotation (green triangles): Previous experimental data ([20], Fig. 5c) for red abalone *H. rufescens* with and without chemotaxis (filled gray triangles: with chemotaxis, open gray triangles: inhibited chemotaxis) and our corresponding simulations (filled green triangles: with chemotaxis, open green triangles: without chemotaxis; mean ± SD), using fertilizability $p_{\mathrm{f}} = 60\%$ in Eq. (5) as single fit parameter. Experiment and simulation again agree reasonably except for the data point without flow $\alpha = 0$ s^{-1}, which corresponds to a different experimental protocol. While the simulations with co-rotation overestimate the reduction of P_{fert} at high shear rate $\alpha > 6$ s^{-1}, these high shear rates are less relevant for the spawning habitat of *H. rufescens*.

A Shear flow around freely-rotating egg and minimal case of ballistic swimmer

For all simulations (except Fig 1A), we use a simple shear flow $\alpha y\, \mathbf{e}_x$ as idealized paradigm for small-scale turbulence. At the relevant shear rates α and typical egg radii $r_{\mathrm{egg}} \sim 100\ \mu$m, the Reynolds number

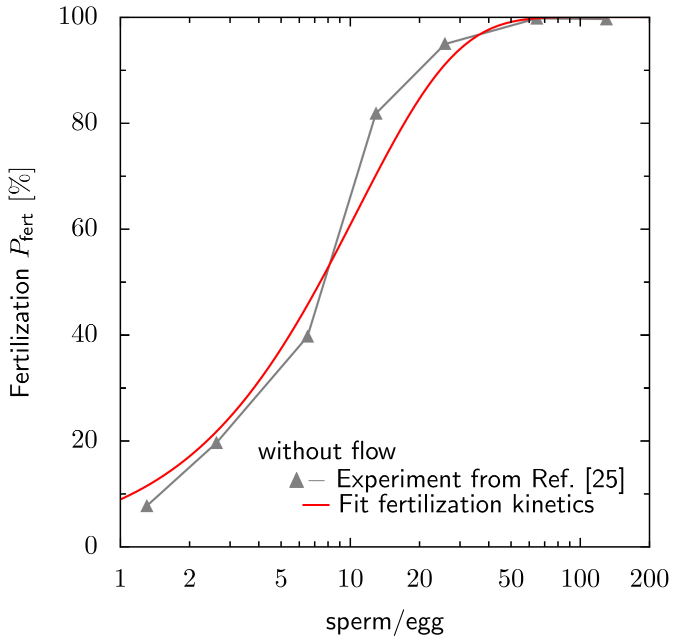

Fig B. Calibration of fertilizability without flow ($\alpha = 0$). Fertilization probability P_{fert} as function of the ratio $\rho_{\text{sperm}}/\rho_{\text{egg}}$ of sperm and egg density in the absence of flow $\alpha = 0\ \text{s}^{-1}$ from experiments with sea urchin *S. purpuratus* ([25], Fig. 4) (filled gray triangles) and fit of fertilization kinetics Eq. (5) (red). From the fit, we obtain $p_{\text{f}} P_{\text{sperm:egg}} \approx 9\%$ for the product of fertilizability p_{f} and encounter probability $P_{\text{sperm:egg}}$. Assuming a ballistic swimmer that is captured at the egg surface (Eq. (5) with $P_{\text{sperm:egg}}(t_{\max}) = 1 - \exp(-q t_{\max})$ and rate $q = \pi r_{\text{egg}}^2 v_h \rho_{\text{egg}} = 0.02\ \text{s}^{-1}$), we find $p_{\text{f}} \approx 10\%$ for exposure time $t_{\max} = 120$ s and $\rho_{\text{egg}} = 1.5 \cdot 10^4\ \text{ml}^{-1}$. This value p_{f} is used in Fig 4 and Fig C.

$\text{Re} = \alpha r_{\text{egg}}^2/\nu \leq 0.1$ is sufficiently small to justify the use of the analytical Stokes equation for viscous flow $\mathbf{v}_{\text{ext}}(\mathbf{r})$. Throughout, we consider the co-moving frame of the egg allowing us to assume that the egg is at the origin $\mathbf{r} = \mathbf{0}$. We introduce dimensionless coordinates $\hat{\mathbf{r}} = \mathbf{r}/r_{\text{egg}}$ and the dimensionless flow field $\hat{\mathbf{v}}_{\text{ext}}(\hat{\mathbf{r}}) = \frac{2\mathbf{v}_{\text{ext}}(\mathbf{r})}{\alpha r_{\text{egg}}}$. The components of this flow field read ([4], Eq. (12))

$$
\begin{aligned}
\hat{v}_{\text{ext},x} &= 2\hat{y} \quad - \hat{y}\left(\left[1+\hat{\Omega}\right]\hat{r}^{-3} + \hat{r}^{-5}\right) - 5\hat{x}^2\hat{y}\left(\hat{r}^{-5} - \hat{r}^{-7}\right) \\
\hat{v}_{\text{ext},y} &= \qquad\quad \hat{x}\left(\left[1+\hat{\Omega}\right]\hat{r}^{-3} - \hat{r}^{-5}\right) - 5\hat{x}\hat{y}^2\left(\hat{r}^{-5} - \hat{r}^{-7}\right) \\
\hat{v}_{\text{ext},z} &= \qquad\qquad\qquad\qquad\qquad\qquad\quad - 5\hat{x}\hat{y}\hat{z}\left(\hat{r}^{-5} - \hat{r}^{-7}\right)
\end{aligned}
\tag{S1}
$$

where no-slip boundary conditions on the surface $|\hat{\mathbf{r}}| = 1$ of the freely-rotating spherical egg are assumed. The egg rotates according to the undisturbed flow vorticity with the dimensionless rotation rate $\hat{\Omega} = -1$, corresponding to an rotation of the egg with angular velocity $\mathbf{\Omega} = -\frac{\alpha}{2}\mathbf{e}_z$.

It is instructive to consider a ballistic swimmer in the above flow field $\mathbf{v}_{\text{ext}}$ as a reference for the analysis of more complicated cases, such as swimmers performing chemotaxis. For instance, without flow or chemotaxis, sperm cells are considered to swim along a straight helix with helix radius r_0 much smaller than the egg radius. These sperm trajectories are well approximated by a ballistic swimmer moving along the helix axis $\mathbf{h}$ with net swimming speed v_h. If the ballistic swimmers and the target eggs (with density ρ_{egg}) are uniformly distributed, the steady-state rate q at which a swimmer hits an egg is given by $q = \pi(r_{\text{egg}} + r_0)^2 v_h \rho_{\text{egg}} \approx \pi r_{\text{egg}}^2 v_h \rho_{\text{egg}}$. If ballistic swimmers become trapped at the egg on encounter, this corresponds to the encounter probability $P_{\text{sperm:egg}}(t) = 1 - \exp(-qt)$ (and fertilization probability P_{fert} according to fertilization kinetics, see Eq. (5)). If ballistic swimmers are additionally

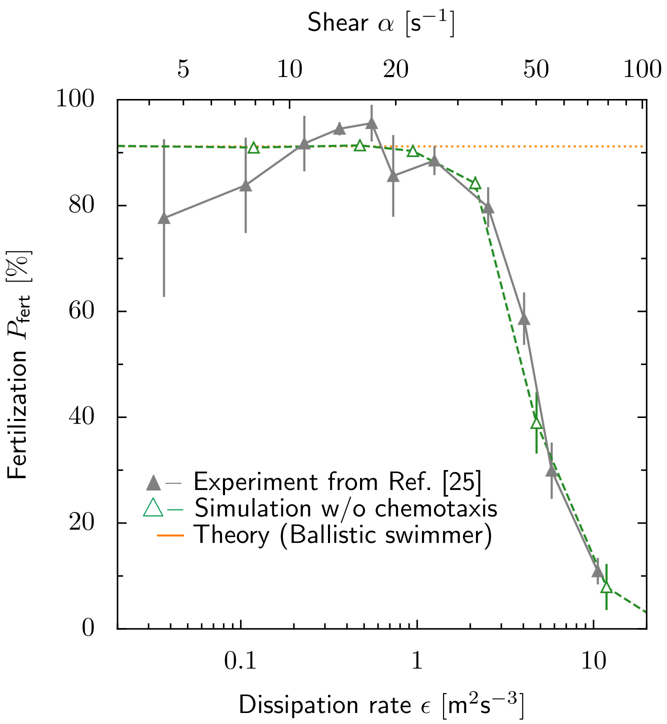

Fig C. Fertilization probability with co-rotation. Fertilization probability $P_{\rm fert}(\alpha)$ as in Fig 4 but with simulations taking into account co-rotation of sperm cells (open green triangles): Previous measurements of fertilization probability $P_{\rm fert}(\epsilon)$ for sea urchin *S. purpuratus* at strong turbulence, characterized by density-normalized dissipation rate ϵ (filled gray triangles) [1, 25] and our corresponding simulations $P_{\rm fert}(\alpha)$ as function of shear rate α (open green triangles, mean ± SD) match well, using a single fit parameter $a = 0.023$ that relates dissipation rate ϵ and typical shear rate α (with the known relationship $\alpha(\epsilon) = a\sqrt{\epsilon/\nu}$ [2, 3]). Analogous to Fig 4, the case of low shear rates is well described by the limit case of a ballistic swimmer in the absence of flow $\alpha = 0\ {\rm s}^{-1}$ (dotted horizontal line, Eq. (5) with $P_{\rm sperm:egg}(t) = 1 - \exp(-qt)$ and rate $q = \pi r_{\rm egg}^2 v_h \rho_{\rm egg}$). The fertilizability $p_{\rm f} = 10\%$ is obtained from an independent experiment [25], see Fig B. From the experimental protocol, we estimate a high background concentration $c_{\rm bg} = 500 - 4000$ nM of chemoattractant, which renders sperm chemotaxis ineffective.

convected by an external fluid flow field $\mathbf{v}_{\rm ext}$, we can characterize q (and thus $P_{\rm sperm:egg}$ and $P_{\rm fert}$) in terms of an universal curve: We introduce the dimensionless parameter $f = \frac{\alpha r_{\rm egg}}{2 v_h}$, which compares shear rate to net swimming speed. The combined velocity field of active swimming and fluid flow is now

$$\mathbf{v}_{\rm ext}(\mathbf{r}) + v_h \mathbf{h} = v_h \left(f \hat{\mathbf{v}}_{\rm ext}(\hat{\mathbf{r}}) + \mathbf{h} \right) = v_h \hat{\mathbf{u}}(\hat{\mathbf{r}}, f, \mathbf{h}) \ . \tag{S2}$$

Note that without co-rotation, $\mathbf{h}$ does not change. Thus, for any $\mathbf{h}$, all possible velocity fields $\hat{\mathbf{u}}$ are given by a single one-parameter family parametrized by f. For each of these fields, the dimensionless rate of swimmers $\hat{q}(f, \mathbf{h})$ reaching the egg from $|\hat{\mathbf{r}}| \gg 1$ specifies the actual rate q for any set of parameters $\alpha, r_{\rm egg}, v_h, \rho_{\rm egg}$ with the same parameter f by

$$q(\alpha, r_{\rm egg}, v_h, \mathbf{h}) = \hat{q}(f, \mathbf{h})\, r_{\rm egg}^2 v_h \rho_{\rm egg} \ . \tag{S3}$$

We obtain a universal curve for q by computing $\hat{q}(f, \mathbf{h})$ numerically for all f and $\mathbf{h}$ and average $\hat{q}(f) = \langle \hat{q}(f, \mathbf{h}) \rangle_{\mathbf{h}}$ over all directions $\mathbf{h}$, see Fig 4 for corresponding $P_{\rm fert}$. A prominent feature of the universal rate is that it vanishes at large shear rates $\hat{q}(f \to \infty) \to 0$. In the absence of flow $\alpha = 0$, we have $\hat{q}(f = 0) = \pi$.

We compute the universal rate $\hat{q}$ efficiently by integrating a uniform grid of initial conditions on the surface of the egg, with $|\hat{\mathbf{r}}| = 1$ at $\hat{t} = 0$, backwards in time according to the velocity field $\hat{\mathbf{u}}$. Each initial condition is integrated until it either returns to the egg $|\hat{\mathbf{r}}|(\hat{t}) = 1$ (fail) or leaves the outer boundaries $\hat{\mathbf{r}}(\hat{t}) = \hat{r}_{\max}$ (success) with $\hat{r}_{\max} \gg 1$. As the flow is volume conserving, the results are independent of the choice of the outer boundary $\hat{r}_{\max}$, as long as $\hat{r}_{\max}$ is sufficiently large to ensure the absence of closed orbits beyond it. We choose $\hat{r}_{\max} = 4$ as numerics show that the in- and outflow on this sphere differs only by 4% between the Stokes flow around the freely-rotating sphere and the undisturbed simple shear flow, for which it is known no closed orbits exist. Based on the intersections with the outer boundary, the flow reaching the egg is interpolated. This is done for a grid of swim directions $\mathbf{h}$. For efficiency, we exploit the symmetries of the Stokes flow $\hat{\mathbf{v}}_{\text{ext}}(\hat{x}, \hat{y}, \hat{z}) \cdot (h_x\mathbf{e}_x + h_y\mathbf{e}_y + h_z\mathbf{e}_z) = \hat{\mathbf{v}}_{\text{ext}}(\hat{x}, \hat{y}, -\hat{z}) \cdot (h_x\mathbf{e}_x + h_y\mathbf{e}_y - h_z\mathbf{e}_z) = \hat{\mathbf{v}}_{\text{ext}}(-\hat{x}, -\hat{y}, \hat{z}) \cdot (-h_x\mathbf{e}_x - h_y\mathbf{e}_y + h_z\mathbf{e}_z)$; thus, it is sufficient to consider $h_z \geq 0$ and $h_y \geq 0$, respectively.

B Equations of motion for navigating sperm cells

We simulate the swimming path $\mathbf{r}(t)$ of a sperm cell in a concentration field $c(\mathbf{r})$ of chemoattractant in the presence of an external fluid flow field $\mathbf{v}_{\text{ext}}(\mathbf{r})$. For this, we extend a previous theory of chemotaxis of marine sperm cells along helical paths [5–8] by incorporating convection and co-rotation by flow: The sperm cell is described in terms of the time-dependent center position $\mathbf{r}(t)$, averaged over one flagellar beat cycle, and the set of ortho-normal vectors $\mathbf{e}_1(t), \mathbf{e}_2(t), \mathbf{e}_3(t)$ of the co-moving coordinate frame, where the vector $\mathbf{e}_1(t)$ points in the direction of active swimming with speed v_0. The equations of motion read

$$\begin{aligned} \dot{\mathbf{r}} &= v_0\mathbf{e}_1 + \mathbf{v}_{\text{ext}}(\mathbf{r}(t)) \ , \\ \dot{\mathbf{e}}_i &= (\boldsymbol{\Omega}_{\text{h}} + \boldsymbol{\Omega}_{\text{f}}) \times \mathbf{e}_i \qquad i = 1, 2, 3 \ , \end{aligned} \tag{S4}$$

The two angular velocities, $\boldsymbol{\Omega}_{\text{h}}$ and $\boldsymbol{\Omega}_{\text{f}}$, describe the rotation of the coordinate frame due to helical chemotaxis and external flow, respectively. For Eq. (S4) a constant swim speed is assumed and motility noise is neglected; the persistence length of sperm swimming paths in the absence of chemoattractant cues was estimated as $3 - 25$ mm [9] which validates this assumption. Note that Eq. (S4) is also valid for time-dependent concentration and flow fields. Note further that the quantitative comparison of the two angular velocities in Eq. (S4) already suggests that the rotation due to external flow is negligible, as the rate of change due to external flow $\Omega_{\text{f}} \sim \alpha = 0 - 1\ \text{s}^{-1}$ (see Eq. (S8)) is always smaller than due to the helical motion $\Omega_{\text{h}} \sim \tau_0 v_0 = 3 - 13\ \text{s}^{-1}$ (see Eq. (S5) and parameters in Table A).

Without external flow or chemotaxis, cells swim along a helical path with constant path curvature $\kappa(t) = \kappa_0$ and torsion $\tau(t) = \tau_0$. The angular velocity $\boldsymbol{\Omega}_{\text{h}}$ is defined by the Frenet-Serret equations

$$\boldsymbol{\Omega}_{\text{h}}(t) = v_0 \left[\tau(t)\mathbf{e}_1(t) + \kappa(t)\mathbf{e}_3(t)\right] \ , \tag{S5}$$

where the coordinate frame $\mathbf{e}_1, \mathbf{e}_2, \mathbf{e}_3$ corresponds to the Frenet-Serret frame of $\mathbf{r}(t)$, i.e., tangent, normal and bi-normal vector. During chemotactic steering, sperm cells dynamically regulate curvature $\kappa(t)$ and torsion $\tau(t)$ of active swimming according to the output $a(t)$ of a chemotactic signaling system

$$\begin{aligned} \kappa(t) &= \kappa_0 - \rho\kappa_0(a - 1) \ , \\ \tau(t) &= \tau_0 + \rho\tau_0(a - 1) \ . \end{aligned} \tag{S6}$$

Here, the sensori-motor gain factor ρ characterizes the amplitude of chemotactic steering responses. The chemotactic signaling system takes as input the local concentration $c(\mathbf{r}(t))$ at the position of the cell

$$\begin{aligned} \mu\dot{a} &= p\left[c_b + c(\mathbf{r}(t))\right] - a \ , \\ \mu\dot{p} &= p\,(1 - a) \ . \end{aligned} \tag{S7}$$

This minimal signaling system comprises sensory adaption with sensitivity threshold c_b and relaxation with time scale μ to a rest state $a = 1$ for any constant stimulus $c(\mathbf{r}(t)) = c_0$. The variable p describes an dynamic sensitivity which is regulated down when the stimulus is high, or regulated up when the

stimulus is low (a loose analogy would be that p corresponds to the opening of our eye's pupils as adaption to brightness). In principle, p and a could have different time-scales [5]. However, equal time-scales automatically ensure that the phase-lag between small-amplitude oscillations of the input signal $c(\mathbf{r}(t))$ and resulting oscillation of the output signal $a(t)$ attains the value $\pi/2$ optimal for helical chemotaxis [6]. This special case is sufficient for the purpose of a minimal model. The gain factor ρ sets the rate of chemotactic steering. While ρ could depend on the chemotactic signal by a feedback mechanism [10], we assume here a constant gain factor $\rho = 5$ for simplicity. The values of all parameters are listed and discussed in Sec G.

We approximate the angular velocity $\mathbf{\Omega}_\mathrm{f}$ for co-rotation by external flow using the Jeffery equation for a small prolate spheroid with major axis along $\mathbf{e}_1$ [11, 12]

$$\begin{aligned}\mathbf{\Omega}_\mathrm{f}(\mathbf{r}) &= \frac{1}{2}\omega(\mathbf{r}) + G\mathbf{e}_1 \times [\mathbf{E}(\mathbf{r}) \cdot \mathbf{e}_1] \ , \\ \omega(\mathbf{r}) &= \mathbf{\nabla} \times \mathbf{v}_\mathrm{ext}(\mathbf{r}) \ , \\ \mathbf{E}(\mathbf{r}) &= \frac{1}{2}\left[\mathbf{\nabla} \otimes \mathbf{v}_\mathrm{ext}(\mathbf{r}) + (\mathbf{\nabla} \otimes \mathbf{v}_\mathrm{ext}(\mathbf{r}))^T\right]\end{aligned} \tag{S8}$$

with the flow vorticity ω, the strain rate tensor $\mathbf{E}$, and a geometric factor $G = \frac{g^2-1}{g^2+1}$, which depends on the aspect ratio $g \geq 1$ of major to minor axis of the spheroid. Together with Eq. (S4), Eq. (S8) describes the cell rotation due the flow, i.e., The first term in the first line of Eq. (S8) describes rotation of a spherical body due to flow vorticity and the second term the correction for non-sperical bodies that can be approximated as spheroids. For a swimming sperm cell, we take the swim direction $\mathbf{e}_1$ as effective major axis, and employ an effective aspect ratio, $g = 5$, reflecting the ratio of the length of the flagellum and a typical beat amplitude [13]. Note that in general instead of $\mathbf{e}_1$, the major axis could be any co-moving vector.

We numerically integrate the equations of motion, i.e., Eqs. (S4,S7), using an Euler scheme with fixed small time step dt. For efficient computation, Rodrigues rotation formula [14] with respect to the co-moving coordinate frame is used to integrate $\mathbf{e}_1, \mathbf{e}_2, \mathbf{e}_3$, resulting in faster computation compared to the algorithm used in [10].

C Analysis of concentration filaments

Turbulent flows cause turbulent mixing of diffusing chemicals and generate filamentous concentration fields. As a minimal model, we simplify the turbulent flow and the filamentous concentration field by the case of a simple shear flow. We consider a spherical egg located at the origin $\mathbf{r} = \mathbf{0}$ releasing chemoattractant with diffusion coefficient D at a constant rate $\dot{Q}$ in the presence of shear flow $\mathbf{v}_\mathrm{ext}(\mathbf{r})$ given by Eq. (S1). We compute the time-dependent concentration field $c(\mathbf{r}, t)$ of chemoattractant numerically using Lagrangian particle tracking, see Sec G. We empirically find that the far-field at distances $r \gg r_\mathrm{egg}$ is well approximated by a generic profile, see Fig 1B for illustration,

$$c(\mathbf{r}, t) = c_0 \exp\left(-k\,|x|\right) \exp\left(-\frac{\left(y - y_0\right)^2 / a_y^2 + z^2}{2\sigma^2}\right) \ , \tag{S9}$$

which describes a concentration filament with time-dependent parameters $c_0(t)$, $k(t)$, a_y, as well as time- and position-dependent variance and midline profiles $\sigma(x, t)$ and $y_0(x, t)$, respectively. This formula for the concentration filament is consistent with results obtained using the analytic solution for an instantaneous point source in a shear flow $\alpha y\, \mathbf{e}_x$, see below. We present and discuss scaling laws for the parameters in the following. While these dependencies are not explicitly required for our theory, they demonstrate the universality of our theory. Finally, we use these scaling laws to quantify how the filaments become longer and thinner with increasing shear rate α.

From numerical simulations, we empirically find the following scaling laws of the parameters from

Eq. (S9)

$$k(t) \sim t^{-\delta_k} \;, \qquad \delta_k = 1.5 - 1.6 \tag{S10}$$

$$c_0(t) \sim t^{-\delta_{c_0}} \;, \qquad \delta_{c_0} = 1.6 - 1.8 \tag{S10b}$$

$$\sigma^2(x,t) = 2D\, t_0(x,t) \;, \qquad t_0 \sim t \tag{S10c}$$

$$t_0(x,t) = p_{0,t_0}(t) + p_{1,t_0}(t)\,|x| + p_{2,t_0}(t)\,|x|^2 \;, \tag{S10d}$$

$$y_0(x,t) = \operatorname{sgn}(x)\, p_{0,y_0}(t) + p_{1,y_0} t^{-\delta_{y_0}} x \;, \qquad \delta_{y_0} = 0.9 - 1.0 \tag{S10e}$$

$$p_{1,y_0} = (1 - 1.4)\alpha^{-\delta_{y_0}} \;, \qquad a_y = 0.5 - 0.6 \tag{S10f}$$

where all parameters except p_{2,t_0} are positive. Note that in a turbulent flow the time t in which the filament is formed may scale with the Kolmogorov time $t \sim \tau_{\mathrm{Kol}}$; in this case σ would scale as the Batchelor length $\sigma \sim \sqrt{D\tau_{\mathrm{Kol}}}$. We also found power-law dependencies for the coefficients $p_{0,t_0}(t)$, $p_{1,t_0}(t)$, and $p_{2,t_0}(t)$. The factor a_y appears to be constant for sufficiently large t. These numerical observations become plausible by analysis of a point source in shear flow. The Fokker-Planck equation for this case can be written in dimensionless form

$$\partial_t c = -\alpha y\, \partial_x c + D \triangle c + \dot{Q}\, \delta(\mathbf{r}) \Rightarrow \partial_{\check{t}} \check{c} = -\check{y}\, \partial_{\check{x}} \check{c} + \triangle \check{c} + \delta(\check{\mathbf{r}}) \tag{S11}$$

by using the Batchelor scale $\sqrt{D\tau_{\mathrm{Kol}}} \sim \sqrt{D/\alpha}$ to re-scale to dimensionless coordinates

$$\check{x} = x\sqrt{\frac{\alpha}{D}} \;, \quad \check{y} = y\sqrt{\frac{\alpha}{D}} \;, \quad \check{t} = t\alpha \;, \quad \check{c} = c\sqrt{\frac{D^3}{\alpha}}\frac{1}{\dot{Q}} \tag{S12}$$

with shear rate α, and release rate $\dot{Q}$ of the source (i.e. $\iiint_{-\infty}^{\infty} \mathrm{d}^3 r\, c(\mathbf{r},t) = \dot{Q}\, t$). Consequently, the solution $\check{c}\left(\check{\mathbf{r}}, \check{t}\right)$ of this equation can be re-scaled to the solution $c(\mathbf{r},t)$ for any set of parameters $\alpha, D, \dot{Q}$. For the above form of the far-field of the filament, this implies that the parameters δ_k, δ_{c_0}, p_{1,y_0} and p_{2,t_0} are universal as they are invariant under the re-scaling Eq. (S12). The analytical solution for the dimensionless concentration $\check{c}$ reads ([15], Eq. (18))

$$\check{c}\left(\check{\mathbf{r}}, \check{t}\right) = \int_0^{\check{t}} \mathrm{d}\check{s}\, \check{G}(\check{\mathbf{r}}, \check{s}) \tag{S13}$$

with Greens function $\check{G}$, i.e., the solution for an instantaneous source at the origin ([16], Eq. (26))

$$\check{G}(\check{\mathbf{r}}, \check{t}) = \frac{\exp\left[-\frac{\left(\check{x} - \frac{1}{2}\check{y}\check{t}\right)^2}{4\check{t}\left(1 + \frac{1}{12}\check{t}^2\right)} - \frac{\check{y}^2 + \check{z}^2}{4\check{t}}\right]}{\left(4\pi\check{t}\right)^{\frac{3}{2}} \sqrt{1 + \frac{1}{12}\check{t}^2}} \;. \tag{S14}$$

While the integral Eq. (S13) cannot be solved analytically, it explains the empirical scaling for the parameters in Eq. (S9) heuristically: It is reasonable to assume that for any $\check{x}$, the parameter $\check{y}_0(\check{t})$ is close to the point $\check{y}_{\max}$ of the maximal concentration of $\check{G}(\check{\mathbf{r}}, \check{t})$. From $\partial_{\check{y}} \check{G}(\check{x}, \check{y}, \check{z} = 0, \check{t})|_{\check{y} = \check{y}_{\max}} = 0$, it follows (for $\check{t} > \sqrt{3}$)

$$\check{y}_0(\check{x}, \check{t}) \approx \check{y}_{\max}(\check{x}, \check{t}) = \frac{3\check{t}\check{x}}{2(\check{t}^2 + 3)} \Rightarrow p_{1,y_0} \approx \frac{3}{2}\check{t}^{-1} \tag{S15}$$

in accordance with the fitted power-law.

The power law $c_0(t) \sim t^{-\frac{3}{2}}$, as suggested by numerics, is plausible since $\check{G}\left(\mathbf{0}, \check{s} \gg 1\right) \sim \check{s}^{-\frac{5}{2}}$, which implies $\check{c}\left(\mathbf{0}, \check{t} \gg 1\right) \sim \int_0^{\check{t}} \mathrm{d}\check{s}\, \check{s}^{-\frac{5}{2}} \sim \check{t}^{-\frac{3}{2}}$.

We introduce the concentration $\check{c}_{\max}$ at the centerline of the filament $\check{c}_{\max}\left(\check{x}, \check{t}\right) = \check{c}\left(\check{x}, \check{y}_0\left(\check{x}, \check{t}\right), \check{z} = 0\right)$. We make the ansatz $\check{c}_{\max}\left(\check{x}, \check{t}\right) = \check{c}_0(\check{t}) \exp\left(-\check{k}\left(\check{t}\right)|\check{x}|\right)$ and derive a power-law for $\check{k}(\check{t})$ in the following.

We expect that $\check{c}_{\max}$ scales proportional to the summed contributions of the Greens functions at the time-dependent centerline, hence we estimate (assuming $\check{t} \gg 1$, we approximate $\check{t}^2 + 3 \to \check{t}^2$, $1 + \check{t}^2/12 \to \check{t}^2/12$ in $\check{G}$)

$$\check{c}_{\max}\left(\check{x}, \check{t}\right) \sim \int_0^{\check{t}} \mathrm{d}\check{s}\, \check{G}(\check{x}, \check{y}_0(\check{x}, \check{s}), \check{z} = 0, \check{s}) \sim \frac{\mathrm{erfc}\left(\sqrt{\frac{3}{4\check{t}^3}}\check{x}\right)}{6\pi\check{x}} \ . \tag{S16}$$

We are interested in the shape of the concentration filament up to a maximal distance $\check{x}_{\max}$ at which the concentration at the centerline decayed to a fraction ι of $\check{c}_0$, $\check{c}_{\max}(\check{x}_{\max}, \check{t}) = \iota c_0(\check{t})$. Any asymptotic tails beyond this distance will likely not be relevant for chemotaxis. Since the decay of $\check{c}_{\max}$ as function of $\check{x}$ is dominated by the numerator in Eq. (S16), the distance $\check{x}_{\max}$ has a time-dependency $\check{x}_{\max}\left(\check{t}\right) \sim \check{t}^{\frac{3}{2}}$ according to the argument of the complementary error-function erfc. Using Eq. (S16), we estimate the time-dependency of $\check{k}(\check{t})$ from

$$-\check{k}(\check{t})\check{x} = \ln\left(\frac{\check{c}_{\max}(\check{x})}{\check{c}_0}\right) \sim \ln\left(\frac{\mathrm{erfc} a}{a}\right) \sim \ln \mathrm{erfc}\,(a) \sim -a \ , \tag{S17}$$

where we introduced $a(\check{x}, \check{t}) = \sqrt{3/4}\ \check{t}^{-\frac{3}{2}}\check{x}$. The crucial point is that for $0 \le \check{x} \le \check{x}_{\max}(\check{t})$, the variable a varies only in a finite interval $0 \le a \le a_{\max}$ with upper bound $a_{\max} = a(\check{x}_{\max}(\check{t}), \check{t}) \sim \check{t}^{-\frac{3}{2}}\check{x}_{\max}(\check{t})$ independent of time $\check{t}$. This allows us to approximate $\ln \mathrm{erfc}\,(a)$ by its Taylor expansion for small $a \ll 1$ in the last step of Eq. (S17). We conclude $\check{k}\left(\check{t}\right) \sim a_{\max}/\check{x}_{\max}(\check{t}) \sim \check{t}^{-\frac{3}{2}}$, as suggested by numerics.

From the above considerations follows that for a constant exposure time $t_{\max}$ the filaments become longer and thinner with increasing shear rate α: From Eq. (S12) follows for the dimensionless exposure time $\check{t}_{\max} = t_{\max}\alpha$ and thus the exponent $\check{k}$ of the dimensionless version of Eq. (S9) scales with $\check{k} \sim (t_{\max}\alpha)^{-\frac{3}{2}}$, which implies according to $\check{k}\check{x} = kx$ a scaling of the effective decay length

$$1/k \sim \alpha\sqrt{Dt_{\max}^3} \ . \tag{S18}$$

This means that for constant exposure time $t_{\max}$ the effective length of the filament increases with shear rate α. Analogously, from $\check{\sigma} \sim \sqrt{t_{\max}\alpha}$ and $\check{c}_0 \sim (t_{\max}\alpha)^{-\frac{3}{2}}$ follows with the re-scaling Eq. (S12) for the effective decay length σ away from the center of the filament and the base concentration c_0

$$\begin{aligned} \sigma &\sim \sqrt{Dt_{\max}} \\ c_0 &\sim \frac{\dot{Q}}{\alpha\left(Dt_{\max}\right)^{\frac{3}{2}}} \ . \end{aligned} \tag{S19}$$

The combination of the effective decay length σ being independent of α and the base concentration c_0 decreasing with increasing α means that the effective width of the filament decreases with increasing α.

D Chemotactic navigation within filament

We derive an effective equation of motion for chemotactic navigation within a typical concentration filament. For simplicity, we initially ignore interaction with the flow and assume that the motion is effectively two-dimensional, i.e., in the xy-plane. Additionally, we employ a two-dimensional version of Eq. (S9) for the concentration filament, setting $a_y = 1$,

$$c(x, y, t) = c_0 \exp\left(-k\,|x|\right) \exp\left(-\frac{(y - y_0)^2}{2\sigma^2}\right) \ . \tag{S20}$$

We introduce the centerline $\mathbf{r}_h(t) = (x(t), y(t), 0)$ of the helical swimming path $\mathbf{r}(t)$, with $\dot{\mathbf{r}}_h = v_h\mathbf{h}$. From a previously established equation for $\mathbf{r}_h$ [5,6], we have

$$\begin{aligned} \dot{x} &= v_h \cos\left(\varphi\right) \ , \qquad \dot{y} = v_h \sin\left(\varphi\right) \ , \\ \dot{\varphi} &= -v_\varphi \frac{|\boldsymbol{\nabla} c|}{c + c_b} \sin\Psi \ , \qquad \Psi = \sphericalangle\left(\boldsymbol{\nabla} c, \mathbf{h}\right) \ , \end{aligned} \tag{S21}$$

describing the alignment of the helix axis $\mathbf{h}$ with the local gradient $\boldsymbol{\nabla} c(\mathbf{r}_h(t))$ of a concentration field $c(\mathbf{r})$. The first equation corresponds to ballistic motion along the helix axis $\mathbf{h}(\varphi) = \cos\varphi\, \mathbf{e}_x + \sin\varphi\, \mathbf{e}_y$ with net swimming speed $v_h = v_0\tau_0/\sqrt{\kappa_0^2+\tau_0^2}$. The second equation describes chemotactic turning of the orientation angle φ, where Ψ denotes the angle enclosed by $\mathbf{h}$ and the local gradient $\boldsymbol{\nabla} c(\mathbf{r_h}(t))$. Here, c_b denotes the adaption threshold and v_φ the chemotactic turning speed, $v_\varphi = \rho v_h \kappa_0^2/\left(\kappa_0^2+\tau_0^2\right)$, $v_\varphi > 0$, with the gain factor ρ and helix parameters κ_0, τ_0. We apply this general theory, Eq. (S21), to the filamentous profile Eq. (S20) and obtain a single dimensionless ODE

$$\ddot{Y} = \Big(\underbrace{-\dot{X}^2 Y}_{\sim\text{oscillator}} + \underbrace{\operatorname{sgn}(X)\ \gamma \dot{X}\dot{Y}}_{\sim\text{damping}} \Big) \underbrace{\frac{c}{c+c_b}}_{\sim\text{dimmer switch}} \tag{S22}$$

with $\dot{X}^2 + \dot{Y}^2 = 1$, $\dot{X} \neq 0$ and a single dimensionless parameter

$$\gamma = k\sigma\sqrt{\frac{v_\varphi}{v_h}} = k\sigma\sqrt{\frac{\rho\kappa_0^2}{\kappa_0^2+\tau_0^2}}\ . \tag{S23}$$

Here, we introduce a characteristic time-scale τ,

$$\tau = \sqrt{\frac{\sigma}{v_\varphi}\cdot\frac{\sigma}{v_h}}\ , \tag{S24}$$

as well as re-scaled coordinates $Y(T) = (y(t)-y_0)/L$, $X(T) = x(t)/L$, $L = v_h\tau$. Dots denote differentiation with respect to re-scaled time $T = t/\tau$, e.g., $\dot{Y} = \mathrm{d}Y/\mathrm{d}T$. The time scale τ is the geometric mean of a characteristic time-scale σ/v_φ of chemotactic steering and a typical time σ/v_h for traversing the cross-sectional width σ of the filament if steering was absent. We have an equation for X analogous to Eq. (S22) (which requires $\dot{Y} \neq 0$ and covers the case $\dot{X} = 0$),

$$\ddot{X} = \left(\dot{X}\dot{Y}Y - \operatorname{sgn}(X)\ \gamma\dot{Y}^2\right)\frac{c}{c+c_b}\ . \tag{S25}$$

The factor $c/(c+c_b)$ in the effective equations of motion, Eqs. (S22, S25), represents a 'dimmer switch' that attenuates chemotactic navigation at low concentration c. Thus, it is reasonable to define the filament as the region where $c(\mathbf{r}) \geq c_b$. In the following, we focus on the dynamics within the filament and approximate $c/(c+c_b) \approx 1$.

The effective equation of motion, Eq. (S22), describes a damped, non-linear oscillator: The first term $\dot{X}^2 Y$ originates from the perpendicular component $\boldsymbol{\nabla}_\perp c = (\mathbf{e}_y \cdot \boldsymbol{\nabla} c)\,\mathbf{e}_y$ of the concentration filament and governs the observed oscillations of sperm cells around the centerline $Y = 0$ of the filament. Heuristically, these oscillations result from sperm cells slowly aligning their helix axis $\mathbf{h}$ parallel to $\boldsymbol{\nabla}_\perp c$ while approaching $Y = 0$. At $Y = 0$, $\boldsymbol{\nabla}_\perp c$ changes its direction, yet sperm cells overshoot due to their finite chemotactic turning speed $v_\varphi < \infty$, before they eventually make a 'U-turn'. The second term $\operatorname{sgn}(X)\,\gamma\dot{X}\dot{Y}$ in Eq. (S22) originates from the exponential decay of concentration along the centerline of the filament and changes the amplitude of the oscillation. In particular, for $\operatorname{sgn}\left(X\dot{X}\right) < 0$, i.e., sperm cells surfing towards the egg, the oscillation is damped, whereas for $\operatorname{sgn}\left(X\dot{X}\right) > 0$, i.e., sperm cells surfing away from the egg, it is amplified. This increase in amplitude can cause sperm cells that are surfing away from the egg to eventually turn around, redirecting them towards the egg. A linear stability analysis of Eq. (S22) around the case of a non-oscillating trajectory $\left(Y,\dot{Y}\right) = (0,0)$ yields the eigenvalues $\omega_{1,2}$ of the Jacobian of the linearization,

$$\omega_{1,2} = \zeta \pm \mathrm{i}\sqrt{1-\zeta^2}\ , \qquad \zeta = \operatorname{sgn}\left(X\dot{X}\right)\frac{\gamma}{2}\ , \tag{S26}$$

which define a harmonic oscillator with dimensionless damping ratio ζ and dimensionless oscillation frequency $\sqrt{1-\zeta^2}$. This analytic result agrees with full simulations of helical chemotaxis in three-dimensional space, see Fig D.

Note that the predicted exponential decay of oscillation amplitude, $\exp(\zeta T) = \exp(\gamma/2 \cdot t/\tau)$, is independent of x since γ/τ is independent of $\sigma^2(x)$. Interestingly, both for Eq. (S22) and full simulations, the angle at which trajectories intersect the centerline $Y = 0$ of the concentration filament is essentially independent of the angle at which they first entered the filament at $Y(c = c_b)$, provided $Y(c = c_b)$ is sufficiently large: For smaller $Y(c = c_b)$, i.e., outer and thus thinner parts of the filament, trajectories will simply pass through the filament, unable to execute a successful turn before they have left the filament again. As the width of the filament decreases away from the egg, this implies that filament surfing will be operative, at most, up to a maximal distance from the egg (which depends on the entry angle), characterized by p_{in}. If we account for convection by shear flow $\mathbf{v}_{\mathrm{ext}} = \alpha y\, \mathbf{e}_x$, Eq. (S25) changes to $\dot{X} \to \dot{X} + \alpha\tau(Y + y_0(X)/L)$. Note that due to $\mathrm{sgn}\,(y_0(x)) = \mathrm{sgn}\,(x)$, sperm cells that surf within the filament towards the egg swim on average against the external flow.

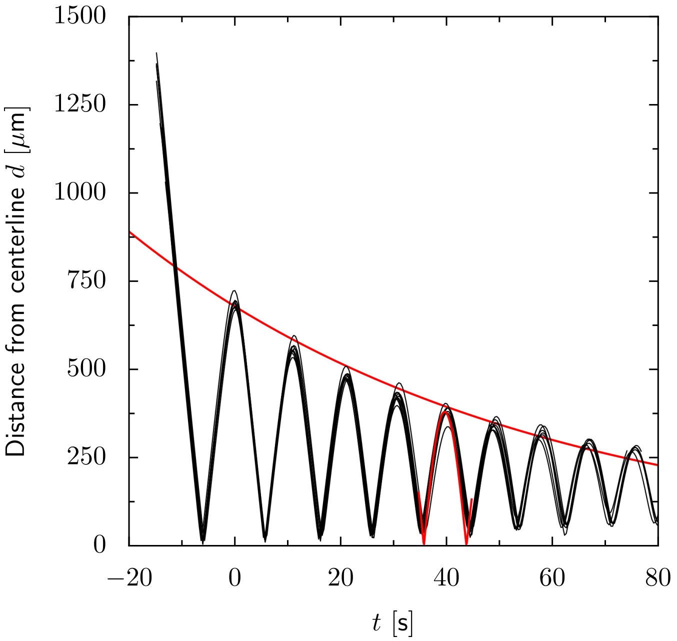

Fig D. Surfing along filaments can be described as damped oscillation. Distance $d = \sqrt{(y - y_0)^2 + z^2}$ from the centerline of concentration filament Eq. (S9) superimposed for $n = 9$ sperm trajectories simulated according to Sec B (black). Trajectories are shown after they entered the surface of the concentration filament, defined by $c(\mathbf{r}(t)) = c_b$, and shifted in time to align the first oscillation peak at $t_0 = 0$. Remarkably, all trajectories display stereotypic oscillations that overlap perfectly, despite the fact that trajectories entered the filament at different x-positions and initial direction angles. The observed damped oscillation are well reproduced by a minimal analytical theory for the centerline of the helical swimming path, which predicts damping ratio and oscillation period (dashed red line, see Eq. (S26)). Parameters as in Fig 1B, corresponding to *A. punctuala*.

E Minimal theory for sperm-egg-encounter probability

We provide an estimate for the encounter probability $P_{\mathrm{sperm:egg}}$, building on the effective equation of motion of the helix axis derived in Sec D. The fertilization probability P_{fert} is obtained then from $P_{\mathrm{sperm:egg}}$ using fertilization kinetics, Eq. (5). For $P_{\mathrm{sperm:egg}}$, we decompose the search problem for the egg into an outer search problem of finding the concentration filament and an inner search problem of surfing along the filament. We obtain (exploiting the symmetry between the two branches of the filament

for $x < 0$ and $x > 0$)

$$P_{\text{sperm:egg}} \approx 2 \int_0^{r_{\max}} \mathrm{d}x\, p_{\text{in}}(x, t_{\max}) \Big[A(x)\rho_{\text{egg}} + S(x) j_{\text{out}} t_{\text{out}}(x, t_{\max}) \Big] \,. \tag{S27}$$

Here, we introduce the following quantities:

- the cross-sectional area $A(x)$ at the position x of the filament, which is defined by $c(\mathbf{r}) \geq c_b$, i.e., $A(x) = \iint_{-\infty}^{\infty} \mathrm{d}y\, \mathrm{d}z\, \Theta(c(x, y, z) - c_b)$, with the Heavyside-function Θ ($\Theta(c > 0) = 1$ and $\Theta(c \leq 0) = 0$),
- the circumference $S(x)$ corresponding to the cross-section,
- the average probability $p_{\text{in}}(x, t_{\max})$ that a trajectory entering the filament at $x > 0$ will surf along it and reach the egg within exposure time $t_{\max}$,
- the mean steady-state flux j_{out} of trajectories arriving at the surface of the filament, and
- the time limit t_{out} for the outer search problem.

These quantities are explained in detail below. The first term in Eq. (S27) accounts for sperm cells found inside the concentration filament already at $t = 0$, assuming a random uniform distribution of initial positions. The second term in Eq. (S27) accounts for trajectories, which first search for the filament and, after encountering the filament, surf along it towards the egg.

We compute the probability $p_{\text{in}}(x, t_{\max})$ of successful inner search numerically using the effective equation of motion for the helix axis Eq. (S22) as function of entry position x and exposure time $t_{\max}$. Specifically, we average over simulations of Eq. (S22) with uniformly distributed initial entry points and isotropic initial directions, i.e., entry angles. In order to account for the ellipsoidal cross-section of the concentration filament with $\sigma_y = \sigma a_y$, $\sigma_z = \sigma$, we average results for σ_y and σ_z. From the successful trajectories, we also obtain the mean travel time t_{in} within the filament, which represents a conditional mean first passage time. Accordingly, we set the maximal time t_{out} allowed for the outer search $t_{\text{out}}(x, t_{\max}) = t_{\max} - t_{\text{in}}(x, t_{\max})$ if $p_{\text{in}} > 0$ and $t_{\text{out}} = 0$ else.

Note that the first term in Eq. (S27) can be written as $V_{\text{eff}}\rho_{\text{egg}}$ with an effective volume $V_{\text{eff}} = 2\int_0^{\infty} \mathrm{d}x\, A(x) p_{\text{in}}(x, t_{\max})$ of the concentration filament, weighted by the probability p_{in} of successful chemotaxis to the egg. This contribution is negligible compared to the second term for long exposure times $t_{\max}$ and low egg densities ρ_{egg}.

The flux j_{out} of trajectories arriving at the surface of the concentration filament can be determined by a fit to $P_{\text{sperm:egg}}(\alpha)$ from simulations at different shear rates α. Alternatively, we can estimate j_{out} by treating sperm cells outside of the filament as ballistic swimmers with net swimming speed v_h and uniformly distributed random positions $\mathbf{r}$ and orientations $\mathbf{h}$ with probability distribution $p_{\text{sperm}}(\mathbf{r}, \mathbf{h}) = \left(\frac{4}{3}\pi(r_{\max}^3 - r_{\text{egg}}^3)\right)^{-1} (4\pi)^{-1} \approx \rho_{\text{egg}}\, (4\pi)^{-1}$. Assuming that the filament is convex, each point on its surface is reached at time t from initial conditions on a surface of a half-sphere with radius $v_h t$. The flux of trajectories with direction $\mathbf{h}$ into the filament at $\mathbf{r}_0$ is $j_{\text{out}}(\mathbf{r}_0, \mathbf{h}) = -\mathbf{n} \cdot v_h \mathbf{h} p_{\text{sperm}}(\mathbf{r}_0, \mathbf{h})$ for $\mathbf{n} \cdot \mathbf{h} < 0$ and $j_{\text{out}}(\mathbf{r}_0, \mathbf{h}) = 0$ else, where $\mathbf{n}$ denotes the outer surface normal vector at $\mathbf{r}_0$. For the constant density $p_{\text{sperm}}(\mathbf{r}_0, \mathbf{h}) = p_{\text{sperm}}$ the total flux of sperm cells into the filament is $j_{\text{out}} = \int_0^{2\pi} \mathrm{d}\varphi \int_0^{\pi/2} \mathrm{d}\theta\, \sin\theta\, j_{\text{out}}(\mathbf{r}_0, \mathbf{h}(\varphi, \theta)) = p_{\text{sperm}} \pi v_h$, where we use spherical coordinates φ, θ with $\mathbf{e}_z = \mathbf{n}$ to express $\mathbf{h}$. Note that an isotropic distribution of orientations $\mathbf{h}$ is a simplification, since co-rotation by flow alters this distribution, see Sec F.

Despite the simplifications made, Eq. (S27) can quantitatively account for the encounter probability in full simulations, see Fig 2. In particular, we find that the numerical fit for $j_{\text{out}} = 0.063\ \text{m}^{-2}\text{s}^{-1}$ is close to our simple estimate for a ballistic swimmer $j_{\text{out}} = \rho_{\text{egg}} v_h / 4 = 0.04\ \text{m}^{-2}\text{s}^{-1}$. Of course, our simple theory has limitations: First, trajectories are three-dimensional, not two-dimensional, and are characterized by oscillations both in y- and z-direction. As a result, sperm trajectories are super-helical, which reduces the effective speed along the filament. Second, our theory does not account for the fact that some sperm cells

may miss the egg on the first attempt, and find it only after reversing their motion in x-direction, which increases the mean time $t_{\rm in}$ to find the egg. Preliminary simulations suggest that the difference between simulations and theory in Fig 2 indeed originate from this effect. Finally, co-rotation is neglected in the simple theory. However, this is justified for $\alpha \ll \tau^{-1}$, see Eq. (S24), i.e., when rotation due to navigation is much faster than co-rotation due to flow. Note that simulations with neither convection nor co-rotation exhibit also an optimal shear rate α^*, but at higher shear rate and different encounter probability. The reason is that convection implies a flow opposing surfing towards the egg, which increases $t_{\rm in}$ compared to the case without convection. Thus, $P_{\rm sperm:egg}$ increases for large α when convection is not included, resulting in a shift of α^*.

For the experiment of Zimmer and Riffell (data reproduced in Fig 3 and Fig A), we estimate a high background concentration of chemotattractant $c_{\rm bg} \sim 4$ nM, see Sec G. Adding a background concentration $c \to c + c_{\rm bg}$ in Eq. (S20) leads to an effective, higher threshold $c_{b,\rm eff} = c_b + c_{\rm bg}$ in Eq. (S22). Consequently, the volume of the filament with sufficiently high concentration $c(\mathbf{r}) \geq c_{b,\rm eff}$ is situated only in the vicinity of the egg. While our far-field theory of filament surfing does not apply directly to this special near-field case, we can make a simple estimate: We assume that sperm cells always swim directly towards the egg within the concentration plume defined by $c(\mathbf{r}) \geq c_{b,\rm eff}$ due to the close-to-spherical shape of the plume. Thus, sperm cells entering the plume at $x_0 = 0$ approach it with net radial speed v_h, as the external flow only convects the sperm cells parallel to the egg surface, see Eq. (S1). A second, alternative calculation applies if sperm cells enter the plume at $x_0 \gg r_{\rm egg}$: In this case, we can estimate the net speed towards the egg by $\dot{x} = \alpha y_0(x) - v_h$. This yields for the distance $x(t)$ from the egg, $x(t) = \frac{v_h}{\alpha b} + \left(x_0 - \frac{v_h}{\alpha b}\right) \exp\left(\alpha b t\right)$ (using $y_0(x) \approx bx$, see Sec C). We use these two limit cases to compute $p_{\rm in}$ and $t_{\rm out}$ for Eq. (S27) and obtain similar fertilization probabilities $P_{\rm fert}(\alpha)$ in both cases. For these limit cases, $P_{\rm fert}(\alpha)$ displays a similar decay as function of α as the simulation results without co-rotation, see Fig 3. In particular, the fitted flux $j_{\rm out} = 4.8 \cdot 10^3\ \mathrm{m^{-2}s^{-1}}$ is consistent with the theoretical value $j_{\rm out} = \rho_{\rm egg} v_h / 4 = 7.5 \cdot 10^3\ \mathrm{m^{-2}s^{-1}}$.

F Analytic solution of Jeffery equation in shear flow

As shear flow is a fundamental paradigm for small-scale turbulence, we present here the analytic solution to the Jeffery equation, Eq. (S8), for particles suspended in simple shear flow. The application to helical swimmers is discussed. The results provide the distribution of helix orientations $\mathbf{h}$ on the periodic boundary used in the simulations, i.e., $p_{\rm sperm}$ in Eq. (6). In particular, the results quantify the common notion that non-spherical swimmers align their major axis parallel to the flow direction. In fact, these swimmers rotate all the time, but with non-constant rotation rate, causing these swimmers to spend more time aligned with the flow axis. Consequently, the time-average of the orientation vector is not zero, but aligned with the flow axis. Note that analytic results for Poiseuille flow can be found in [17,18].

For simple shear flow $\mathbf{v}_{\rm ext} = \alpha y\, \mathbf{e}_x$, the dynamics of the unit vector $\mathbf{e}$ along the major axis of a prolate spheroid, i.e., $\dot{\mathbf{e}} = \mathbf{\Omega}_{\rm f} \times \mathbf{e}$ with $\mathbf{\Omega}_{\rm f}$ given by Eq. (S8), can be rewritten in terms of spherical coordinates $0 \leq \theta \leq \pi, 0 \leq \varphi \leq 2\pi$ of $\mathbf{e} = (\sin\theta\cos\varphi, \sin\theta\sin\varphi, \cos\theta)$

$$\begin{aligned} \dot{\theta} &= \frac{\alpha G}{4} \sin 2\theta \sin 2\varphi \ , \\ \dot{\varphi} &= \frac{\alpha}{2} \left[G \cos 2\varphi - 1 \right] \ . \end{aligned} \tag{S28}$$

The range $1 \leq g < \infty$ of the aspect ratio g (with $g = 0$ for a sphere and $g \to \infty$ for an infinitesimal thin rod) implies $0 \leq G < 1$ for the geometric factor G. The dynamics of the polar angle $\varphi(t)$ is independent of the azimuthal angle $\theta(t)$. By integration, we find

$$\varphi(t) = \arctan\left[\frac{G-1}{\sqrt{1-G^2}} \tan\left(\Psi(t)\right) \right] \tag{S29}$$

with short-hand

$$\Psi(t) = \frac{\alpha t}{2}\sqrt{1-G^2} + \arctan\left[\frac{\sqrt{1-G^2}}{G-1}\tan\varphi_0\right] \tag{S30}$$

and initial condition $\varphi(0) = \varphi_0$. Note that $\dot{\varphi} \leq 0$, i.e., $-\frac{\alpha}{2}(1+G) \leq \dot{\varphi} \leq -\frac{\alpha}{2}(1-G)$. Hence, the polar angle $\varphi(t)$ rotates clockwise with period

$$T = \frac{4\pi}{\alpha\sqrt{1-G^2}} \tag{S31}$$

with $T \geq 4\pi/\alpha$. Substituting Eq. (S29) for $\varphi(t)$ into Eq. (S28), we find

$$\theta(t) = \operatorname{arccot}\left[\cot(\theta_0)\sqrt{\frac{1+G\cos[2\Psi(0)]}{1+G\cos[2\Psi(t)]}}\right] \tag{S32}$$

with initial condition $\theta(0) = \theta_0$.

We also compute the density $\rho_{\mathbf{e}}(\theta, \varphi)$ of directions for an ensemble of ballistic microswimmers obeying Eq. (S28). The distribution of polar angles $\rho_\varphi(\varphi)$ is proportional to $1/|\dot{\varphi}|$

$$\rho_\varphi(\varphi) = \frac{\sqrt{1-G^2}}{4\pi\left[1-G\left(1-2\sin^2\varphi\right)\right]} \ . \tag{S33}$$

This density has two maxima, at $\varphi_+ = 0$ and $\varphi_+ = \pi$, and two minima at $\varphi_- = \pm\pi/2$, resulting in a density range $\rho_\varphi(\varphi_-) \leq \rho_\varphi \leq \rho_\varphi(\varphi_+)$ with $\rho_\varphi(\varphi_\pm) = (4\pi)^{-1}\left[(1+G)/(1-G)\right]^{\pm 1/2}$.

In order to derive the full density $\rho_{\mathbf{e}}(\theta, \varphi)$, we use an alternative scheme to solve the continuity equation, inspired by the method of characteristics. Effectively, an ordinary differential equation (ODE) and a system of ODEs are solved instead of one partial differential equation (PDE). The dynamics of $\mathbf{e}$ correspond to a flow $\mathbf{w}(\mathbf{e})$ on the unit sphere. The continuity equation for a density $\rho_{\mathbf{e}}(\mathbf{e}, t)$ in an arbitrary flow field $\mathbf{w}(\mathbf{e}, t)$ reads

$$\partial_t \rho_{\mathbf{e}}(\mathbf{e}, t) = -\boldsymbol{\nabla}\cdot\left[\rho_{\mathbf{e}}(\mathbf{e}, t)\mathbf{w}(\mathbf{e}, t)\right] \ . \tag{S34}$$

Instead of solving directly for the density $\rho_{\mathbf{e}}(\mathbf{e}, t)$ in the laboratory frame, we can first solve for the density $\breve{\rho}_{\mathbf{e}}(t; \mathbf{e}_0)$ in a co-moving frame

$$\rho_{\mathbf{e}}(\mathbf{e}, t) = \breve{\rho}_{\mathbf{e}}\left(t; \breve{\mathbf{e}}(-t, \mathbf{e})\right) \ , \tag{S35}$$

where $\breve{\mathbf{e}}(t; \mathbf{e}_0)$ is the trajectory starting at $\breve{\mathbf{e}}(0; \mathbf{e}_0) = \mathbf{e}_0$ and following the flow $\dot{\breve{\mathbf{e}}} = \mathbf{w}(\breve{\mathbf{e}}, t)$. We obtain $\breve{\rho}_{\mathbf{e}}(t; \mathbf{e}_0)$ from the rewritten continuity equation

$$\dot{\breve{\rho}}_{\mathbf{e}}(t; \mathbf{e}_0) = -\breve{\rho}_{\mathbf{e}}(t; \mathbf{e}_0)\,\boldsymbol{\nabla}\cdot\mathbf{w}\left(\breve{\mathbf{e}}(t; \mathbf{e}_0), t\right) \ . \tag{S36}$$

Applying this scheme to Eq. (S28) with flow $\mathbf{w}(\theta, \varphi) = \dot{\theta}\mathbf{e}_\theta + \sin\theta\dot{\varphi}\mathbf{e}_\varphi$ on the unit sphere and using the solutions $\theta(t), \varphi(t)$ from Eqs. (S29,S32) yields

$$\breve{\rho}_{\mathbf{e}}(t; \theta_0, \varphi_0) = C_0(\theta_0, \varphi_0)\left[\cot^2(\theta_0)\left(1+G\cos[2\Psi(0)]\right) + \left(1+G\cos[2\Psi(t)]\right)\right]^{\frac{3}{2}} \ , \tag{S37}$$

where the pre-factor $C_0(\theta_0, \varphi_0)$ is defined by the initial conditions. For our simulations, we use an initially uniform distribution such that $\breve{\rho}_{\mathbf{e}}(0, \theta_0, \varphi_0) = (4\pi)^{-1}$. Switching notation to $\breve{\theta}(t, \theta_0, \varphi_0) = \theta(t)$ and $\breve{\varphi}(t, \varphi_0) = \varphi(t)$, the density $\rho_{\mathbf{e}}$ follows

$$\rho_{\mathbf{e}}(\theta, \varphi, t) = \breve{\rho}_{\mathbf{e}}\left(t; \breve{\theta}(-t, \theta, \varphi), \breve{\varphi}(-t, \varphi)\right) \ . \tag{S38}$$

While $\rho_{\mathbf{e}}$ is periodic in time with period T by Eq. (S31), we can compute a time-average over one period, starting with a uniform distribution of directions $\mathbf{e}$ at $t = 0$. The time-averaged density displays a maximum at the axis of flow $\mathbf{e} = \pm\mathbf{e}_x$ and a minimum at the shear axis $\mathbf{e} = \pm\mathbf{e}_y$. These extrema vanish for a sphere ($G = 0$) and become more pronounced with increasing G.

While the above results are derived for the case of a suspended particle, numerical simulations show that they also approximately apply to the centerline $\mathbf{r}_h(t)$ of a helical swimmer with helix axis $\mathbf{h}$ (without chemotaxis) if we use an effective aspect ratio g_{eff}. Specifically, the dynamics of the helix axis $\mathbf{h}$ resembles the above solutions with a smaller aspect ratio $1 \leq g_{\mathrm{eff}} \leq g$. This approximation is valid for small times t and at small α, i.e., as long as the helix period is much smaller than the period $T(g)$. For instance, we fit $g_{\mathrm{eff}} = 1.3 \pm 0.1$ ($G_{\mathrm{eff}} = 0.26$) for the sea urchin helix parameters and $g = 5$ ($G = 0.92$). This effective parameter is a result of averaging the instantaneous co-rotation for the swimming direction $\mathbf{e}_1$ with parameter G over one period of helical swimming. Generally, g_{eff} depends on the angle between $\mathbf{h}$ and $\mathbf{e}_1$. For larger α, complicated behavior of $\mathbf{h}$ is observed with limit cycles and stable fixed points, which is consistent with recent results for Jeffery equation in perturbed shear flow [19]. We use the value g_{eff} in all simulations to determine the periodic boundary conditions at the boundary of the simulation domain.

G Choice of parameters

Parameters used throughout the three simulation scenarios (*Arabacia punctuala* for Fig 1B, Fig 2, Fig D, *Strongylocentrotus purpuratus* from [1, 21, 22, 25] for Fig 4, Fig C and Fig B, *Haliotis rufescens* from [13, 20] for Fig 3 and Fig A) are listed in Table A and discussed in the following.

Mean path curvature $\kappa_0 = 0.065\ \mu\mathrm{m}^{-1}$ and mean path torsion $\tau_0 = 0.067\ \mu\mathrm{m}^{-1}$ of the helical paths are set according to three-dimensional tracking of *A. punctuala* sperm cells [8]. Three-dimensional tracking for *S. purpuratus* give similar values [23], though with larger error intervals. Moreover, the sperm morphology for *A. punctuala* [8, 10], *S. purpuratus* [24], and *H. rufescens* [26] is similar, which justifies the use of the same helix parameters for all three species. Likewise, the effective aspect ratio $g = 5$ between major and minor axis of a sperm cell, i.e., length of flagellum divided by typical beat amplitude, suggested for *H. rufescens* [13] is employed for all three species in the Jeffery equation Eq. (S8). We observe that simulation results are largely independent of the precise value of g. The signaling time-scale $\mu = 1/\left(v_0\sqrt{\kappa_0^2 + \tau_0^2}\right)$ is chosen to ensure the optimal phase-lag between concentration input $c(\mathbf{r}(t))$ and motor response $a(t)$ [5, 27], see Eq. (S7), consistent with experimental observations [8]. For all three species, the gain factor is set as $\rho = 5$, corresponding to the mean of the values used in [10]. This value reproduces typical bending rates of helical swimming paths as observed in experiments [8]. The threshold of sensory adaption $c_b = 10$ pM is chosen as suggested in [28]. At the concentration c_b, about 20 chemoattractant molecules would diffuse to a sperm cell during one helical turn. Note that sea urchin sperm cells respond to single chemoattractant molecules [29]; the change in intra-cellular calcium concentration caused by the binding of chemoattractant molecules as function of stimulus strength becomes sub-linear already for chemoattractant concentrations on the order of c_b [28]. For *A. punctuala*, other parameters were also tested, i.e., $\rho = 2$ and $c_b = 1$ pM, which yielded qualitatively similar simulation results and again agreement of theory and simulations. Note that the experimental protocol used in [20] for *H. rufescens* results in a substantial background concentration of chemoattractant, which we estimate as $c_{\mathrm{bg}} \sim 4$ nM (experiments are conducted $10 - 30$ min after spawning at a high density of eggs $\rho_{\mathrm{egg}} = 10^3\ \mathrm{ml}^{-1}$ with the known release rate $\dot{Q} = 0.18$ fmol min^{-1} of chemoattractant [30]). According to our theory, such a background concentration causes effectively a higher sensitivity threshold $c_{b,\mathrm{eff}} = c_b + c_{\mathrm{bg}}$ (see Sec E), which may the be reason for the higher behavioral threshold 300 pM observed in [20]. In the case of *S. purpuratus*, we estimate an even higher background concentration, $c_{\mathrm{bg}} \sim 500 - 4000$ nM, which renders chemotaxis ineffective. For this estimate, we use that experiments were conducted $1 - 8$ h after spawning at a high egg density $\rho_{\mathrm{egg}} = 1.5 \cdot 10^4\ \mathrm{ml}^{-1}$ [21, 25] and assume a release rate $\dot{Q} = 0.46$ fmol min^{-1} of chemoattractant as for *A. punctuala* [28].

For the swimming speed v_0 of sperm cells along helical paths for both sea urchin species, we use the measured value $v_0 = 200\ \mu\mathrm{m\ s}^{-1}$ from [8]. Note that some experiments effectively measure the net swimming speed along the helix axis $v_h = v_0\tau_0/\sqrt{\kappa_0^2 + \tau_0^2}$, which is smaller than v_0. For *H. rufescens*, we

use the speed v_h measured during the same experiment [20]. Note that this experiment also indicated chemokinesis, i.e., higher swimming speeds at elevated chemoattractant concentration, an effect which we neglect here for simplicity.

For *A. punctuala*, we use the diffusion coefficient $D = 239\ \mu\text{m}^2\text{s}^{-1}$ and release rate $\dot{Q} = 0.46$ fmol min^{-1} of chemoattractant [28]. For this simulation, we assume a low egg density $\rho_{\text{egg}} = 10^{-3}\ \text{ml}^{-1}$, which yields the radius $r_{\max} = 6 \cdot 10^4\ \mu$m of the outer boundary centered around the egg according to $\rho_{\text{egg}} = \left(4\pi r_{\max}^3/3\right)^{-1}$. For this reference case, the filament is completely included inside the simulation domain for all considered shear rates α. The exposure time $t_{\max} = 360$ s is chosen comparable to the experiment in [25], where $t_{\max} = 120$ s. While for this work the exposure time $t_{\max}$ is set by the protocol of the considered experiment, in a generic turbulent flow $t_{\max}$ corresponds to the time-scale of flow changes, i.e. scale with the Kolmogorov time $t_{\max} \sim \tau_{\text{Kol}}$. For comparison with the experiments with *S. purpuratus* and *H. rufescens*, the radius $r_{\max}$ is computed directly from the stated egg density $\rho_{\text{egg}} = \left(4\pi r_{\max}^3/3\right)^{-1}$. From the 5 vol% solution with $r_{\text{egg}} = 40 - 55\ \mu$m ([21], pg. 161), we infer a range $\rho_{\text{egg}} = 0.9 - 3.4 \cdot 10^4\ \text{ml}^{-1}$ for the experiments with *S. purpuratus*. This estimate already takes into account that, according to the experimental protocol, the above egg solution is mixed 9 : 1 with sperm solution [21, 25]. Likewise, from the range of sperm densities $\rho_{\text{sperm}} = 1.9 - 3.1 \cdot 10^6\ \text{ml}^{-1}$ in Fig. 4 of [25] and the estimate $\rho_{\text{sperm}} = 4 \cdot 10^6\ \text{ml}^{-1}$ from pg. 59 of [21], both before 9 : 1-dilution, we infer a final concentration $\rho_{\text{sperm}} = 3.9 \cdot 10^5\ \text{ml}^{-1}$. We use the kinematic viscosity $\nu = 10^{-6}\ \text{m}^2\text{s}^{-1}$ of sea water at room temperature.

H Numerical Simulation

The equations of motion are integrated using an Euler scheme with fixed time step $\mathrm{d}t$. For all time integrations, a time step $\mathrm{d}t = 10^{-3}$ s is used. Integration with smaller $\mathrm{d}t = 10^{-4}$ s for some test cases gave consistent results. The number N_{sperm} of sperm cells simulated in each case is 10^5, except for *S. purpuratus*, where $N_{\text{sperm}} = 10^4$ is used.

The concentration field is computed from Lagrangian particle tracking with Euler-Maruyama method for the Fokker-Planck equation

$$\partial_t c = -\boldsymbol{\nabla} \cdot \mathbf{v}_{\text{ext}}\ c + D \bigtriangleup c \tag{S39}$$

with $\mathbf{v}_{\text{ext}}$ from Eq. (S1). Test particles were released at random points of the surface of sphere of radius r_{egg} located at the origin. In total, we used $4 \cdot 10^6$ test particles, which corresponds effectively to $1.6 \cdot 10^7$ particles by exploiting symmetries of the flow field. Concentrations are evaluated on a cubic $50 \times 50 \times 50$ grid, spanning in each dimension from $-r_{\max}$ to $r_{\max}$, and then interpolated by a spline interpolation of order 3. This grid is sufficiently fine to resolve the details of the concentration filaments. The rapid convergence to a near-steady state allows to use a static concentration field corresponding to exposure time $t_{\max}$ for each simulation. We checked for test cases that full simulations with time-varying concentration field do not yield different results.

The implementation of an unsteady shear flow for a shear rate α used as illustration in Fig 1A is inspired by [31]: We use the flow field $\mathbf{v}_{\text{ext}}(\mathbf{r}, t) = \alpha'(r, t)\ \left[\mathbf{r} \cdot \mathbf{e}'_y(t)\right]\ \mathbf{e}'_x(t)$, where the shear axis $\mathbf{e}'_y(t)$ and the flow axis $\mathbf{e}'_x(t)$ are subject to a three-dimensional random walk on the unit sphere with rotational diffusion coefficient $D_{\text{rot}} = \pi\alpha$. The shear rate profile is given by $\alpha'(r, t) = \sqrt{2}\ \alpha \sin(2\pi t/T_\alpha)\ h(r)$. The shear rate $\alpha'(r, t)$ decays as $h(r)$ with distance r away from the center. This decay $h(r)$ mimics the decay of velocity from the center of a vortex. We use the decay of an Lamb-Oseen vortex $h(r) = \left(\frac{r_{\text{core}}}{r}\right)^2 \left(1 - \exp\left[-\left(\frac{r}{r_{\text{core}}}\right)^2\right]\right)$, employing the Burger radius r_{B} of a Burger vortex as core radius $r_{\text{core}} = r_{\text{B}}$, where $r_{\text{B}} = K\eta_{\text{Kol}} \approx K\sqrt{\frac{\nu}{\alpha}}$ with $K = 7.1$ [3, 32, 33]. The shear rate $\alpha'(r, t)$ oscillates in time with root-mean-square amplitude $\sqrt{\frac{1}{T_\alpha}\int_0^{T_\alpha} \mathrm{d}t\, \alpha'(r, t)^2} = h(r)\alpha$ and period $T_\alpha = \frac{r_B^2}{2\nu}$, corresponding to the time scale of decay of a Burger vortex.

Parameter			Sea urchin [8] ($A.$ $punctuala$) Fig 1, Fig 2, Fig D	Sea urchin [1, 21, 22, 25] ($S.$ $purpuratus$) Fig 4, Fig B, Fig C	Red abalone [13, 20] ($H.$ $rufescens$) Fig 3, Fig A
path curvature	κ_0	$\left[\mu\text{m}^{-1}\right]$	0.065		
path torsion	τ_0	$\left[\mu\text{m}^{-1}\right]$	0.067		
helix radius	r_0	$[\mu\text{m}]$		$\kappa_0 / \left(\kappa_0^2 + \tau_0^2\right) \approx 7$	
gain factor	ρ			5	
threshold of sensory adaption	c_b	[pM]		10	
signaling time-scale	μ	[s]		$\left(v_0 \sqrt{\kappa_0^2 + \tau_0^2}\right)^{-1}$	
sperm aspect ratio	g			5	
swimming speed	v_0	$\left[\mu\text{m s}^{-1}\right]$	200		42
net speed along helix axis	v_h	$\left[\mu\text{m s}^{-1}\right]$	145		30
chemoattractant release rate	$\dot{Q}$	$\left[\text{fmol min}^{-1}\right]$	0.46		0.18
diffusion coefficient	D	$\left[\mu\text{m}^2\text{s}^{-1}\right]$	239		660
egg radius	r_{egg}	$[\mu\text{m}]$	100	50	108
egg density	ρ_{egg}	$\left[\text{ml}^{-1}\right]$	10^{-3}	$1.5 \cdot 10^4$	10^3
boundary radius	$r_{\max}$	$[\mu\text{m}]$	$6 \cdot 10^4$	240	620
sperm density	ρ_{sperm}	$\left[\text{ml}^{-1}\right]$	–	$3.9 \cdot 10^5$	10^4
exposure time	$t_{\max}$	[s]	360	120	15
background concentration	c_{bg}	[nM]	–	$500 - 4000$	4
fertilizability (fit)	p_{f}		–	10%	60%

Table A. List of parameters used or obtained for the three scenarios. See text for discussion and further parameters.

I Parameter study

In order to demonstrate the sensitivity of the quantitative results, shown in Fig 2 of the main text, on the parameters, we computed the encounter probability $P_{\text{sperm:egg}}(\alpha)$ for a range of exposure times $t_{\max}$, egg densities ρ_{egg} expressed in terms of boundary radii $r_{\max}$, threshold of sensory adaption c_b, and gain factors ρ as shown in Fig E, Fig F, Fig G, and Fig H, respectively. In all cases, there is a pronounced optimum present at some intermediate shear rate α^*, where the position of the optimum α^* is only slightly affected by the parameter variations. The parameters mostly affect the height of the optimum, in particular $t_{\max}$ and $r_{\max}$, and its ratio to the flow-less $\alpha = 0$ case, see Table B. This suggests that the existence of an optimum is quite insensitive to parameter variations, i.e., regardless of how the parameters are adapted, fertilization is optimal at an intermediate shear flow for a broad physiological range of parameter values.

For each parameter study all parameters but one are kept constant on the values reported for *A. punctuala* in Table A. The only exception are $t_{\max}$ and $r_{\max}$ whose base values are lowered to $t_{\max} = 90$ s and $r_{\max} = 15$ mm for numerical efficiency, i.e., the blue dots in Fig E, Fig F, Fig H, and Fig G correspond always to the same parameters. The increase of the exposure time $t_{\max}$ in Fig E from 45 to 90 s causes only a slight decrease of the optimal shear rate α^* from 0.3 to 0.1 s^{-1}, but increases the absolute encounter probability $P_{\text{sperm:egg}}(\alpha^*)$ by an order of magnitude. Such an increase of $P_{\text{sperm:egg}}(\alpha^*)$ is also observed for the increase of the egg density, i.e., the decrease of the boundary radius from 30 to 10 mm in Fig F. These increases are in accordance with the simple argument that longer search time or smaller search volume increases the chances of finding the egg. The advantage of the optimum to the flow-less case $P_{\text{sperm:egg}}(\alpha^*)/P_{\text{sperm:egg}}(\alpha = 0)$ varies for both parameters between a factor 2 and 14, see Table B. In contrast, the variation of c_b and ρ hardly affects the optimum in terms of α^* and $P_{\text{sperm:egg}}(\alpha^*)$ but rather alters the probability in the absence of flow: Increasing c_b or decreasing ρ increases $P_{\text{sperm:egg}}(\alpha = 0\ \text{s}^{-1})$. This is probably an effect of signal-noise, originating from the computed concentration field which, due to the very nature of Lagrangian particle tracking, can exhibit low signal-to-noise ratio at low concentrations, i.e. at the surface of the concentration plume. (Note that our model does not explicitely account for sensing noise [34].) This noise results in an effective reflection of incoming sperm trajectories at the surface of the plume for increasing sensitivity of the concentration measurement, expressed by c_b, or increasing reaction to signal stimulus, expressed by ρ, see also discussion in [10,35]. The effect is expected to be much smaller for concentration filaments at $\alpha > 0\ \text{s}^{-1}$ as the concentration gradient towards the center of the filament is higher and thus the signal-to-noise ratio generally higher as for a concentration plume solely established by diffusion in the flow-less case.

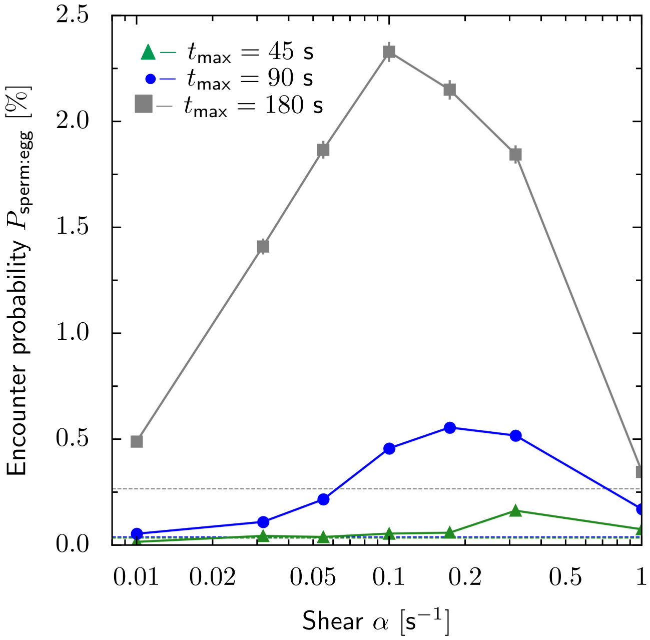

Fig E. Flow-dependent sperm-egg encounter probability for different exposure times t_{max}. Encounter probabilities $P_{sperm:egg}(\alpha)$ as function of external shear rate α for three values of sperm-egg exposure time t_{max} obtained from simulations with co-rotation. (symbols according to legend, mean ± SD; flow-less results $P_{sperm:egg}(\alpha = 0\ s^{-1})$ displayed by dashed horizontal lines in respective color). Parameters taken for *A. punctuala*, see Table A, except boundary radius $r_{max} = 15$ mm for numerical efficiency.

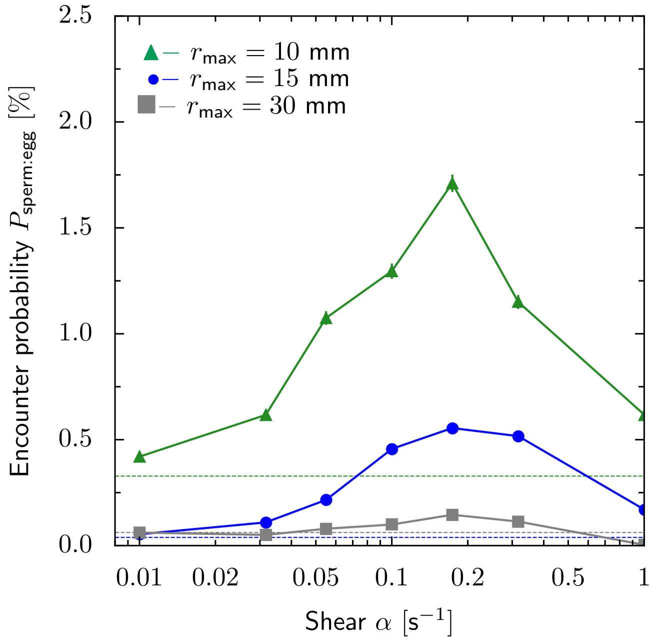

Fig F. Flow-dependent sperm-egg encounter probability for different egg densities ρ_{egg}. Analogous to Fig E, yet for three different values of boundary radius r_{max}, corresponding to three different egg densities ρ_{egg} according to $\rho_{egg} = \left(4\pi r_{max}^3/3\right)^{-1}$. (For all three curves $t_{max} = 90$ s, thus blue curve identical to blue curve in Fig E.)

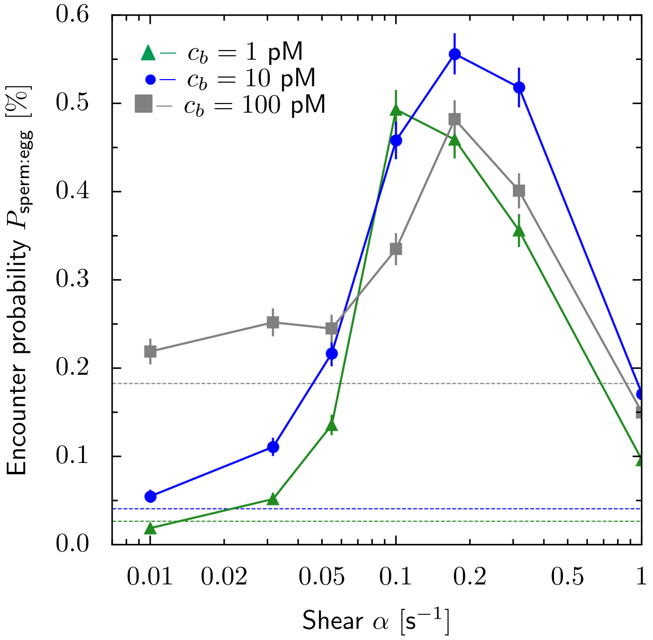

Fig G. Flow-dependent sperm-egg encounter probability for different thresholds of sensory adaption c_b. Analogous to Fig E, yet for three different values of threshold c_b. (For all three curves $t_{max} = 90$ s and $r_{max} = 15$ mm, thus blue curve identical to blue curve in Fig E.)

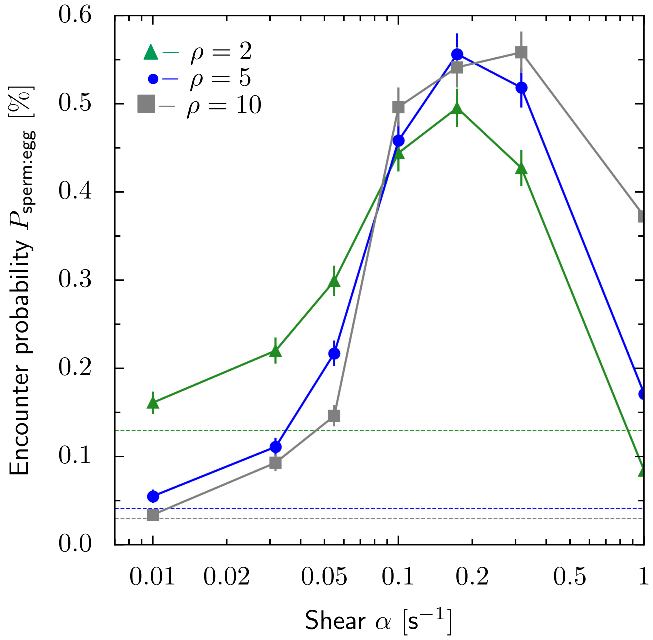

Fig H. Flow-dependent sperm-egg encounter probability for different gain factors ρ. Analogous to Fig E, yet for three different values of gain factor ρ. (For all three curves $t_{\max} = 90$ s and $r_{\max} = 15$ mm, thus blue curve identical to blue curve in Fig E.)

Parameter	$P_{\text{sperm:egg}}(\alpha^*)/P_{\text{sperm:egg}}(\alpha = 0)$		
Sperm-egg exposure time: $t_{\max} = 45$ s, 90 s, 180 s	4.6	13.6	8.7
Boundary radius setting egg density: $r_{\max} = 10$ mm, 15 mm, 30 mm	5.2	13.6	2.3
Threshold of sensory adaptation: $c_b = 1$ pM, 10 pM, 100 pM	18.3	13.6	2.6
Chemotactic gain factor: $\rho = 2$, 5, 10	3.8	13.6	18.6

Table B. Relative amplitude of optimum in sperm-egg encounter probability. Ratios of encounter probability $P_{\text{sperm:egg}}(\alpha = \alpha^*)$ at optimal shear rate α^* normalized by encounter probability $P_{\text{sperm:egg}}(\alpha = 0)$ in the absence of flow for parameter study displayed in Fig E, Fig F, Fig G, and Fig H.

References

1. Gaylord B. Hydrodynamic Context for Considering Turbulence Impacts on External Fertilization. Biol Bull. 2008;214(3):315–318. doi:10.2307/25470672.

2. Lazier JRN, Mann KH. Turbulence and the Diffusive Layers around Small Organisms. Deep-Sea Res I. 1989;36(11):1721–1733. doi:10.1016/0198-0149(89)90068-X.

3. Jumars PA, Trowbridge JH, Boss E, Karp-Boss L. Turbulence-Plankton Interactions: A New Cartoon. Mar Ecol. 2009;30(2):133–150. doi:10.1111/j.1439-0485.2009.00288.x.

4. Mikulencak DR, Morris JF. Stationary Shear Flow around Fixed and Free Bodies at Finite Reynolds Number. J Fluid Mech. 2004;520:215–242. doi:10.1017/S0022112004001648.

5. Friedrich BM, Jülicher F. Chemotaxis of sperm cells. Proc Natl Acad Sci USA. 2007;104(33):13256–13261. doi:10.1073/pnas.0703530104.

6. Friedrich BM, Jülicher F. Steering Chiral Swimmers along Noisy Helical Paths. Phys Rev Lett. 2009;103(6):068102. doi:10.1103/PhysRevLett.103.068102.

7. Alvarez L, Friedrich BM, Gompper G, Kaupp UB. The Computational Sperm Cell. Trends in Cell Biology. 2014;24(3):198–207. doi:10.1016/j.tcb.2013.10.004.

8. Jikeli JF, Alvarez L, Friedrich BM, Wilson LG, Pascal R, Colin R, et al. Sperm Navigation along Helical Paths in 3D Chemoattractant Landscapes. Nat Commun. 2015;6:7985. doi:10.1038/ncomms8985.

9. Friedrich BM. Search along persistent random walks. Phys Biol. 2008;5(2):026007. doi:10.1088/1478-3975/5/2/026007.

10. Kromer JA, Märcker S, Lange S, Baier C, Friedrich BM. Decision making improves sperm chemotaxis in the presence of noise. PLoS Comput Biol. 2018;14(4):e1006109. doi:10.1371/journal.pcbi.1006109.

11. Jeffery GB. The Motion of Ellipsoidal Particles Immersed in a Viscous Fluid. Proc R Soc Lon A. 1922;102(715):161–179. doi:10.1098/rspa.1922.0078.

12. Pedley TJ, Kessler JO. Hydrodynamic Phenomena in Suspensions of Swimming Microorganisms. Annu Rev Fluid Mech. 1992;24(1):313–358. doi:10.1146/annurev.fl.24.010192.001525.

13. Riffell JA, Zimmer RK. Sex and flow: the consequences of fluid shear for sperm–egg interactions. J Exp Biol. 2007;210(20):3644–3660. doi:10.1242/jeb.008516.

14. Rodrigues O. Des lois géométriques qui régissent les déplacements d'un système solide dans l'espace, et de la variation des coordonnées provenant de ces déplacements considérés indépendamment des causes qui peuvent les produire. J Math Pures Appl. 1840; p. 380–440.

15. Frankel NA, Acrivos A. Heat and Mass Transfer from Small Spheres and Cylinders Freely Suspended in Shear Flow. Phys Fluids. 1968;11(9):1913–1918. doi:10.1063/1.1692218.

16. Elrick DE. Source Functions for Diffusion in Uniform Shear Flow. Aust J Phys. 1962;15(3):283–288. doi:10.1071/ph620283.

17. Zöttl A, Stark H. Nonlinear Dynamics of a Microswimmer in Poiseuille Flow. Phys Rev Lett. 2012;108(21):218104. doi:10.1103/PhysRevLett.108.218104.

18. Zöttl A, Stark H. Periodic and Quasiperiodic Motion of an Elongated Microswimmer in Poiseuille Flow. Eur Phys J E. 2013;36(1):4. doi:10.1140/epje/i2013-13004-5.

19. Słomka J, Alcolombri U, Secchi E, Stocker R, Fernandez VI. Encounter rates between bacteria and small sinking particles. New J Phys. 2020;22(4):043016. doi:10.1088/1367-2630/ab73c9.

20. Zimmer RK, Riffell JA. Sperm Chemotaxis, Fluid Shear, and the Evolution of Sexual Reproduction. Proc Natl Acad Sci USA. 2011;108(32):13200–13205. doi:10.1073/pnas.1018666108.

21. Mead KS. Sex in the surf zone: the effect of hydrodynamic shear stress on the fertilization and early development of free-spawning invertebrates [Ph.D. thesis]. Stanford University; 1996. Available from: `https://dennylab.stanford.edu/sites/g/files/sbiybj6166/f/mead_kristina_thesis.pdf`.

22. Denny MW, Nelson EK, Mead KS. Revised Estimates of the Effects of Turbulence on Fertilization in the Purple Sea Urchin, *Strongylocentrotus purpuratus*. Biol Bull. 2002;203(3):275–277. doi:10.2307/1543570.

23. Corkidi G, Taboada B, Wood CD, Guerrero A, Darszon A. Tracking sperm in three-dimensions. Biochem Biophys Res Commun. 2008;373(1):125–129. doi:10.1016/j.bbrc.2008.05.189.

24. Alvarez L, Dai L, Friedrich BM, Kashikar ND, Gregor I, Pascal R, et al. The rate of change in Ca^{2+} concentration controls sperm chemotaxis. J Cell Biol. 2012;196(5):653–663. doi:10.1083/jcb.201106096.

25. Mead KS, Denny MW. The Effects of Hydrodynamic Shear Stress on Fertilization and Early Development of the Purple Sea Urchin *Strongylocentrotus purpuratus*. Biol Bull. 1995;188(1):46–56. doi:10.2307/1542066.

26. Horst Gvd, Bennett M, Bishop JDD. CASA in invertebrates. Reproduction Fertility and Development. 2018;30(6):907–918. doi:10.1071/RD17470.

27. Friedrich BM. Chemotaxis of Sperm cells [Ph.D. thesis]. TU Dresden; 2008. Available from: https://cfaed.tu-dresden.de/files/user/bfriedrich/papers/friedrich_chemotaxis_sperm_cells_phd_2009.pdf.

28. Kashikar ND, Alvarez L, Seifert R, Gregor I, Jäckle O, Beyermann M, et al. Temporal Sampling, Resetting, and Adaptation Orchestrate Gradient Sensing in Sperm. J Cell Biol. 2012;198(6):1075–1091. doi:10.1083/jcb.201204024.

29. Pichlo M, Bungert-Plümke S, Weyand I, Seifert R, Bönigk W, Strünker T, et al. High density and ligand affinity confer ultrasensitive signal detection by a guanylyl cyclase chemoreceptor. J Cell Biol. 2014;206(4):541–557. doi:10.1083/jcb.201402027.

30. Riffell JA, Krug PJ, Zimmer RK. The ecological and evolutionary consequences of sperm chemoattraction. Proc Natl Acad Sci USA. 2004;101(13):4501–4506. doi:10.1073/pnas.0304594101.

31. Bell AF, Crimaldi JP. Effect of Steady and Unsteady Flow on Chemoattractant Plume Formation and Sperm Taxis. J Marine Syst. 2015;148:236–248. doi:10.1016/j.jmarsys.2015.03.008.

32. Hatakeyama N, Kambe T. Statistical Laws of Random Strained Vortices in Turbulence. Phys Rev Lett. 1997;79(7):1257–1260. doi:10.1103/PhysRevLett.79.1257.

33. Webster DR, Young DL. A Laboratory Realization of the Burgers' Vortex Cartoon of Turbulence-Plankton Interactions. Limnol Oceanogr: Methods. 2015;13(2):92–102. doi:10.1002/lom3.10010.

34. Berg HC, Purcell EM. Physics of chemoreception. Biophysical Journal. 1977;20(2):193–219. doi:10.1016/S0006-3495(77)85544-6.

35. Kromer JA, de la Cruz N, Friedrich BM. Chemokinetic Scattering, Trapping, and Avoidance of Active Brownian Particles. Phys Rev Lett. 2020;124(11):118101. doi:10.1103/PhysRevLett.124.118101.
